# Computational Study Reveals the Role of Water Molecules in the Inhibition Mechanism of LAT1 by 1,2,3-Dithiazoles

**DOI:** 10.1021/acs.jcim.1c01012

**Published:** 2021-11-17

**Authors:** Mario Prejanò, Isabella Romeo, Maria Antonietta La Serra, Nino Russo, Tiziana Marino

**Affiliations:** Department of Chemistry and Chemical Technologies, University of Calabria, 87036 Arcavacata di Rende, Cosenza, Italy

## Abstract

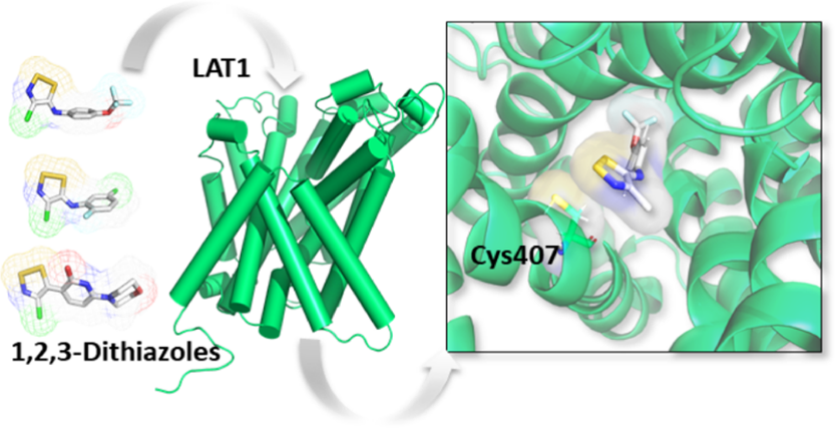

The L-type amino acid transporter LAT1, involved in many biological processes including the overexpression of some tumors, is considered a potential pharmacological target. The 1,2,3-Dithiazole scaffold was predicted to inhibit LAT1 by the formation of an intermolecular disulfide bond with the thiolate group of cysteine(s). As a result of the identification of these irreversible covalent inhibitors, we decided to deeply investigate the recognition stage and the covalent interaction, characterizing the chemical structures of the selected ligands. With the aim to provide new insights into the access of the ligands to the binding pocket and to reveal the residues involved in the inhibition, we performed docking, molecular dynamics simulations, and density functional theory-based investigation of three 1,2,3-dithiazoles against LAT1. Our computational analysis further highlighted the crucial role played by water molecules in the inhibition mechanism. The results here presented are consistent with experimental observations and provide insights that can be helpful for the rational design of new-to-come LAT1’s inhibitors.

## Introduction

The l-type amino acid transporter LAT1 (SLC7A5) is a neutral amino acid transmembrane transporter that acts in cooperation with the glycoprotein CD98 (SLC3A2) for the intracellular amino acid exchange. Indeed, LAT1 is engaged by the CD98 heavy chain, also known as CD98HC or 4F2hc, through a disulfide bond association, but also interacts with the membrane, allowing for the transport activity of the complex by both extracellular and intracellular sides.^1^ For that reason, LAT1 plays a pivotal role in cell metabolism and growth owing to its engagement in the distribution of essential amino acids (His, Met, Leu, Ile, Val, Phe, Tyr, and Trp) to the placenta and blood–brain barrier.^2^ Furthermore, its presence is required in cells with a continuous flux of amino acids that is independent of sodium and pH.^3^ In particular, it has been shown that LAT1 has higher specificity for phenylalanine.^4^ Besides its key role of the antiporter of amino acids and other substrates such as l-DOPA, melphalan, and gabapentin, LAT1 is also involved in the permeation of thyroid hormones, drugs, and hormone precursors.^5,6^ Additionally, because it has been revealed that LAT1 is overexpressed in different tumor types, thus resulting in the reduction of leucine uptake and cell proliferation,^7^ LAT1 can be considered a marker of malignancy and consequently a hopeful anticancer target to inhibit.^8^ Several efforts have been made to identify potent ligands through *in silico* and *in vitro* screenings. Among the possible strategies to block the activity of LAT1, the rational drug design of covalent inhibitors may represent a reasonable way of suppressing malignant cancers. The covalent inhibitors show nonequilibrium binding kinetics, restricting the competition in the presence of a large amount of endogenous substrates for target binding, and their inhibitory activity can be achieved with low doses and protracted duration of action with respect to the classical inhibitors.^9,10^

**Scheme 1:**
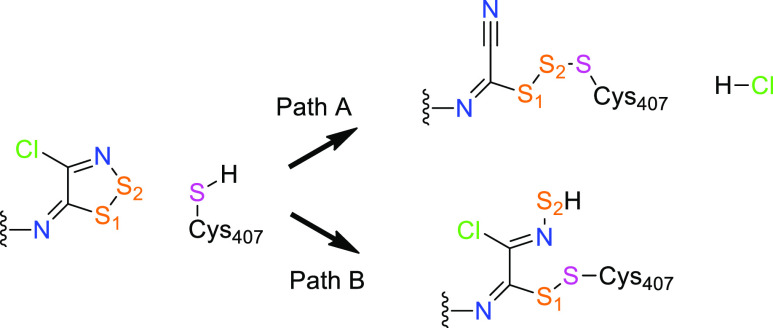
Inhibition of LAT1 by 1,2,3 Dithiazole Compounds

Generally, the common feature for the reaction resulting in the transporter inhibition is characterized by the presence of an electrophilic moiety suffering the nucleophilic attack by the thiolate group of cysteine into the binding pocket. Indeed, the LAT1 binding site includes Cys335 and Cys407 residues that can form disulfides inhibiting in an irreversible way. Recent research has revealed that dithiazole and dithiazine-based covalent inhibitors, in the order of sub-micromolar range, can inactivate LAT1 and, in particular, two of them provoked cell death in high-LAT1-expressing cervical cancer cells (SiHa), thus showing a cytotoxicity activity of these compounds.^12^ This suggested that the inhibition can be induced by the formation of the disulfide or trisulfide intermediate, as reported in Scheme 1. Very interestingly, the contribution of water molecules in the inhibition mechanism has not yet been considered, due to the absence of indications from the available structural data, mainly obtained from 3D homology models and cryo-electron microscopy (cryo-EM) structures.^3,11,12^ Further support from the theoretical chemistry can contribute to elucidate this aspect providing deeper insights on the solvent contribution, which can play a crucial role in both binding and inhibition phases.^13^

**Scheme 2:**
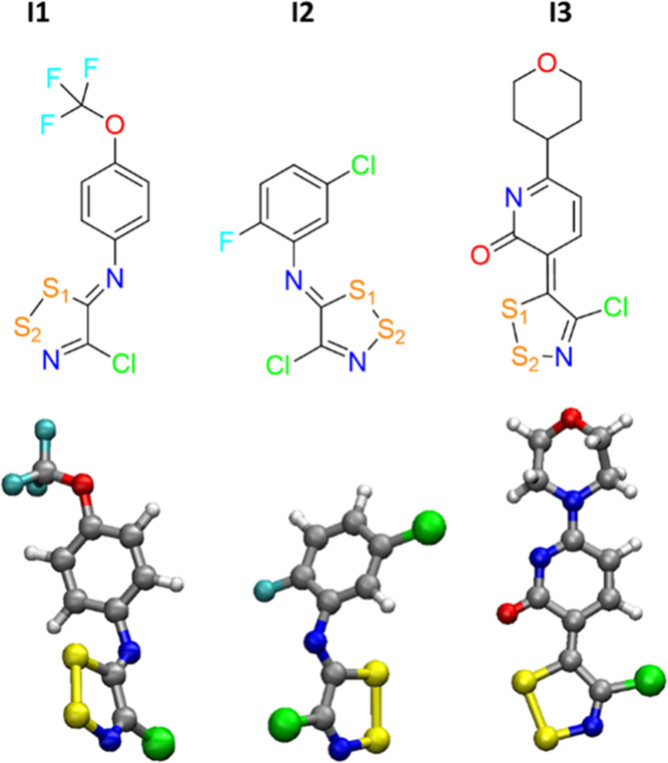
2D (Top) and 3D (Bottom) Structures of the Three 1,2,3-Dithiazoles Considered in the Study; **I1** and **I2** Are the Most Effective Compounds against LAT1 and **I3** is Used as the Negative Control for Computational Investigations

Among the inhibitors tested so far,^12^ the **I1** and **I2** compounds (Scheme 2) exhibited promising anticancer activity, suggesting a strong LAT1 affinity. Indeed, the available experimental data revealed that these two compounds strongly inhibited the LAT1 transporter with IC_50_ values of 0.98 ± 0.10 and 0.89 ± 0.33 μM, respectively. The half saturation constants *K*_i_, calculated from the experiments, were 0.76 ± 0.27 and 1.13 ± 0.41 μM for **I1** and **I2**, respectively.

Considering the pharmacological importance of LAT1, further insights are required to better characterize the transporter and inhibition processes. Because mechanistic understanding of the inhibition of LAT1, not yet clear, is necessary to drive a rational design of its inhibitor, in the present work we present the results of all-atoms molecular dynamics (MDs) simulations and density functional theory (DFT) calculations devoted to identify the most viable mechanism from a thermodynamic and kinetic point of view of three 1,2,3-dithiazoles (**I1**, **I2**, and **I3** sketched in Scheme 2) into the LAT1 binding pocket.

Above **I1** and **I2**, the species **I3** (see Scheme 2) has been taken into account as the negative control in the experimental work, because at 100 μM concentration, the inhibitor showed a LAT1 inhibition lower than 30%,^12^ and for this reason, it has been further adopted in our investigation.

The analysis of the trajectories revealed the presence of water molecules inside the protein channel and in proximity to the reacting centre Cys407-dithiazol ring. The role of water molecules in the reaction, not considered in previous studies, has then been also highlighted *via* full quantum mechanics calculation based on the cluster approach. The two proposed inhibition mechanisms already shown in Scheme 1 were investigated.^12^ Taken together, these results can provide better rationalization of the experimental data, with insights that can be useful for rational design of more efficient LAT1 inhibitors.

## Computational Methods

### Classical MD

Our computational procedure, as usual,^14−17^ requires preliminary all-atoms MD on *apo-form* and complexed LAT1 coupled to the DFT study. In order to take this into account, starting from the 3D homology model of LAT1,^11^ MD simulation was carried out on the free protein. The homology model was characterized by residues 45–488.

The protonation states of the ionisable residues at physiological pH were evaluated by means of the H++ method (see Supporting Information for details).^18^ Antechamber and *parmchk* modules of AMBER16^19^ were used for generating preparatory files. The LAT1 structure was oriented according to the OPM database before being embedded into a POPC lipid bilayer to mimic physiological conditions through CHARMM-GUI webserver.^20^

The free protein was protonated, and counter ions were added appropriately to make the total charge zero. To fully solvate the system, additional water molecules were placed in an orthorhombic box with a buffer of 10 Å. The FF14SB force field for the system and POPC phospholipids^21^ were used,^22^ along with the TIP3P model^23^ for water. The solvated structure was first minimized by applying positional harmonic restraints on all atoms (50 kcal mol^–1^ Å^2^) using 5000 steps of steepest descent^24^ followed by 5000 steps of the conjugate gradient. In the second minimization step, the whole system was released without any restraint and then a progressive heating phase was carried out from 0 to 300 K for 50 ps, followed by 50 ps at 300 K using the Langevin thermostat in the *NVT* ensemble. The production phase was performed for 100 ns of MDs under the following conditions: integration step of the 2 fs coupling SHAKE algorithm and *NPT* ensemble at 1 bar pressure using the Berendsen barostat^25^ with a time constant τp = 2.0 ps. The particle mesh Ewald summation method^26^ was employed for the electrostatic potential, and the long-range electrostatic interactions were calculated with a 12 Å cut-off distance. With the aim to find different representative conformations of the system, root-mean square deviation (RMSD)-based clustering of the whole trajectory according to the relaxed complex scheme (RCS) docking protocol was performed.^27,28^ After removing overall rotations and translations by RMS-fitting the Cα atoms’ positions of the trajectory, the average linkage clustering algorithm, implemented in *cpptraj*, was applied to identify 10 representative conformations for further investigation. These structural insights allowed both the local and global flexibility of the protein and decreased the computational cost for the RCS docking procedure.^29,30^

With the aim to investigate the possible binding modes and interactions of 1,2,3-dithiazoles with the LAT1 active site, molecular docking simulations were carried out using AutoDock version 4.2.^31^ Each representative structure was prepared by assigning atom types and adding Gasteiger charges.^32^ The docking area was established using AutoGrid. A box of dimension 40 × 40 × 40 Å was chosen, and the grid box was centered to the sulfur atom of Cys407; a 0.375 Å grid point spacing was calculated for each atom types. The Lamarckian genetic algorithm was used for ligand conformational searching. The docking conditions were as follows: one docking simulation for each of the 10 representative protein structures, population size of 150, random starting position and conformation, local search rate of 0.6, and 2 500 000 energy evaluations. Final docked poses were clustered using a RMSD tolerance of 1.5 Å. The three best docked poses for each ligand in complex with LAT1 were chosen for further 100 ns of MDs. They were selected by applying the geometrical filter such as the distance between the sulfur atom of the ligands and the Cys thiol at position 407 and according to the stronger theoretical binding affinity. For each ligand, the restrained electrostatic potential^33^ charges were calculated by using the HF/6-31* level of theory^34^ in Gaussian 09 D.01.^35^ All parameters are reported in the Supporting Information. After the preparations of the complexes, 100 ns of MDs was performed using the above-mentioned conditions. The whole trajectories were analyzed in terms of the RMSD and root mean square fluctuation (RMSF). It is worth nothing that in the course of our investigation, many cryoEM solved structures of LAT1 (resolution > 2.7 Å), mainly in complex with other biologically relevant macromolecules, have been released.^3,36^ The choice of homology modeling represents a good starting point for studying the intrinsic conformational properties of the LAT1 protein in the absence of other agents, which could influence the structural behavior. Moreover, the secondary structure of LAT1 in the homology model is highly conserved, compared to the available experimental structure, as can be observed by the superposition between the model and many available cryo-EM structures, as given in the Supporting Information (Figure S1). Additional 50 ns of cMD on the *apo-form* LAT1 model, starting from the resolved cryo-EM structure of LAT1, lies in complex with 4F2hc and the JX-078 inhibitor (PDB7DSL),^36^ and relevant structural variations were not observed (see Supporting Information for the detailed description of protocol and the main results).

### Quantum Mechanics

**Figure 1:**
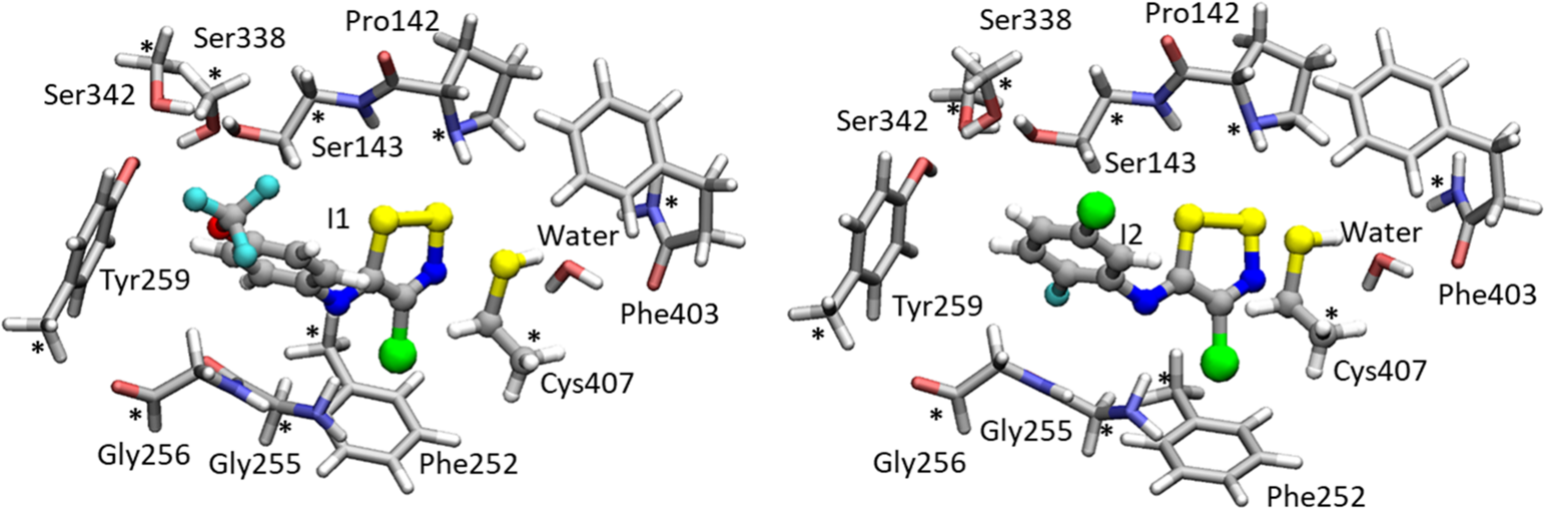
Models adopted for the inhibition mechanism of LAT1 by **I1** (left) and **I2** (right).

A chemically representative model of LAT1 inhibitors was built up from docking of both **I1** and **I2** species in the available 3D homology model of LAT1.^11^ Each model consists of the inhibitor, Cys407, which is the main target of inhibition, and amino acid residues interacting with the 1,2,3-thiazole (Pro142, Ser143, Phe252, Gly255, Gly256, Tyr259, Ser338, Ser342, and Phe403), as depicted in Figure 1. The amino acids were truncated, as shown in Figure 1, and the carbon in which truncation occurred was kept frozen during optimizations to avoid artificial movements of the residues.^37−39^ One water molecule was also explicitly included in the model due to its crucial role as evidenced by our MDs. The final sizes of the model are 137 and 136 atoms for **I1** and **I2**, respectively, and the total charge is 0.

We consider that for the proposal before mentioned and for our consolidated experience on the topic,^40^ the cluster model size represents an adequate combination of chemical and computational accuracy and efficiency to describe the chemical events underlying the inhibition. However, the cluster model is not without limitations^41−43^ but able to discriminate among different mechanisms providing information to be exploited to generate new candidate LAT1 inhibitors.^12^

All calculations were performed with the Gaussian 09 D.01.^35^ The B3LYP-D3^44−47^ combined with the 6-31+G(d,p) basis set for all atoms was used for the optimization of all stationary points intercepted along the potential energy surface. The effect of the protein environment was calculated via single-point energy calculations on the B3LYP-D3/6-31+G(d,p) using the solvation model PCM^48^ with a dielectric constant of ε = 4, in line with recent studies.^49−51^ The zero-point energies were obtained through analytical frequency calculations on stationary points at the same level of theory of geometry optimizations. Single-point calculations were performed with the extended 6-311+G(2d, 2p) basis set to obtain more accurate electronic energies. Finally, natural bond orbital (NBO) charges were calculated at the same level of theory.^52^

## Results and Discussion

### MDs of Unbound- and Bound-Form of LAT1

The first part of the study has been dedicated to classical MD aimed at a better understanding the potential conformational changes of LAT-1; our attention was focused on the structural stability of both apo- and inhibitor-bound proteins, the noncovalent interaction of the inhibitors and the hydrogen bond networks in the targeted region.

To do this, a careful analysis of the properties, such as RMSD, RMSF, hydrogen bonding, and water occupancy near amino acid residues of the binding site was performed.

RMSD values of the LAT-1 backbone and side-chain atom positions for the 100 ns MD simulation time are plotted in Figure S2. The *apo-form* protein appears stable during the majority of the simulation time, meanwhile the RMSD trend of side-chain (4.97 Å) atoms shows higher values with respect to that of the backbone (3.68 Å). These structural trends are in agreement with the requirement of a no rigid binding site of LAT-1 that facilitates its cellular uptake of hydrophobic amino acids and the trans-membrane segment (TM) α-helix opening and the accompanying side-chain reorientations. Previous studies have assumed that LAT1 exhibits a transport mechanism comparable to that of AdiC.^53^ MD reveals that the reorganizations in the simultaneous way of different gating residues led to the conformational change from the outward-open to the inward-open structure in the homolog AdiC.^54^ Analyzing the MDs, it is possible to observe that the conformational rearrangements mainly affect residues surrounding the binding pocket. In particular, Phe252 exhibits a different orientation of the phenyl group of the side chain, thus confirming its role as a proximal gate of LAT1 for the substrate entry to the binding site, as already proved by site-directed mutagenesis studies.^55^

**Figure 2:**
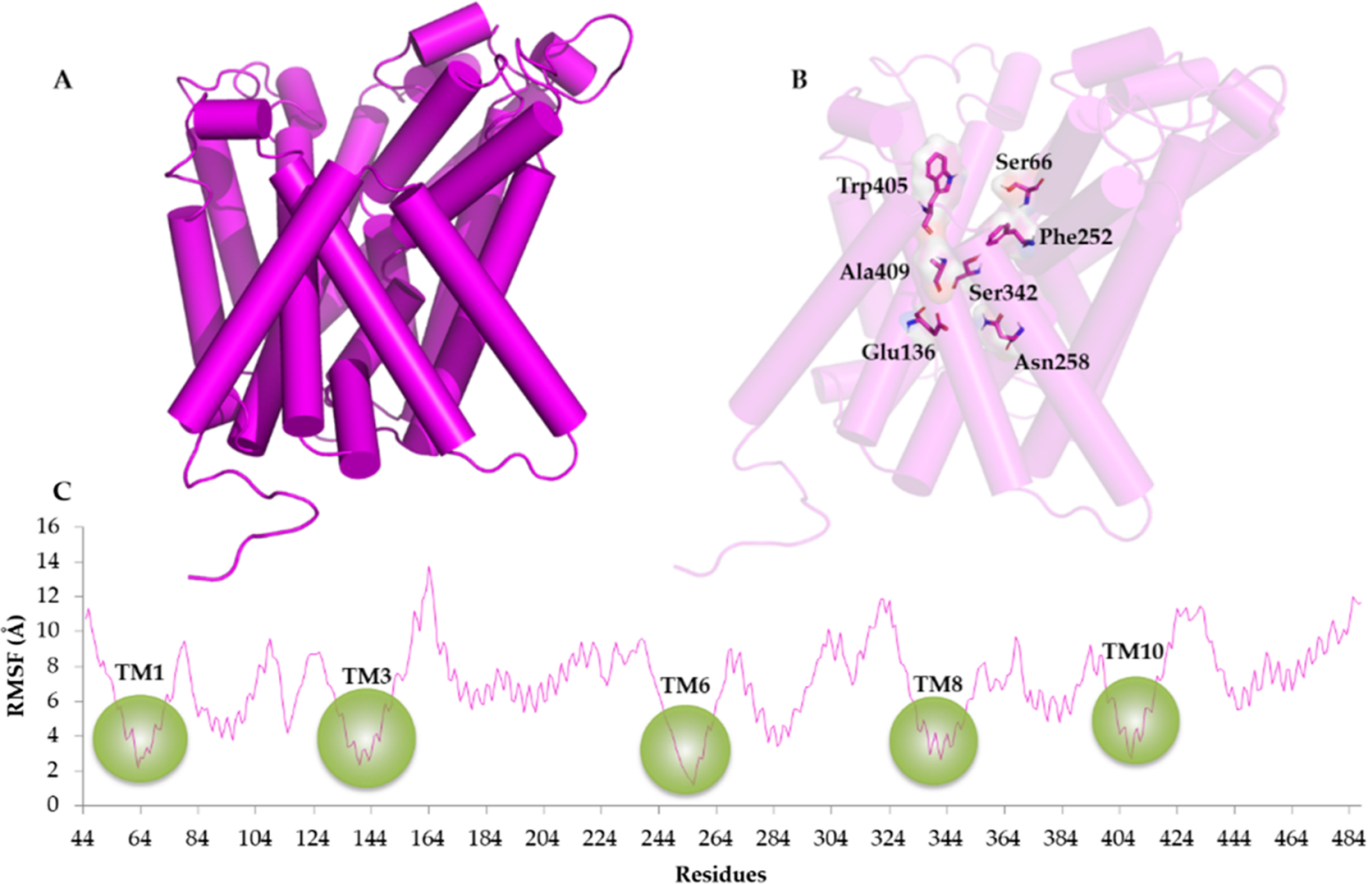
(A) Front view of LAT1 reported as magenta cartoon. (B) Front view of LAT1 showing functionally essential gate residues. The gate residues (Ser66, Glu136, Asn258, Ser342, Trp405, and Ala409) are shown as magenta carbon sticks. (C) RMSF plot of LAT-1 protein residues (magenta line). Green cycles individuate the transmembrane regions (TM1, TM3, TM6, TM8, and TM10) of the inner layer of LAT1.

RMSF analysis (reported in Figure 2), which evidences the residues (Ser66, Glu136, Asn258, Ser342, Trp405, and Ala409) located in the highly conserved region TMs TM1, TM3, TM6, TM8, and TM10 (underlined by green cycle in Figure 2), presents a rigid structural profile with respect to the other regions, allowing for the entry of the substrate.

**Figure 3:**
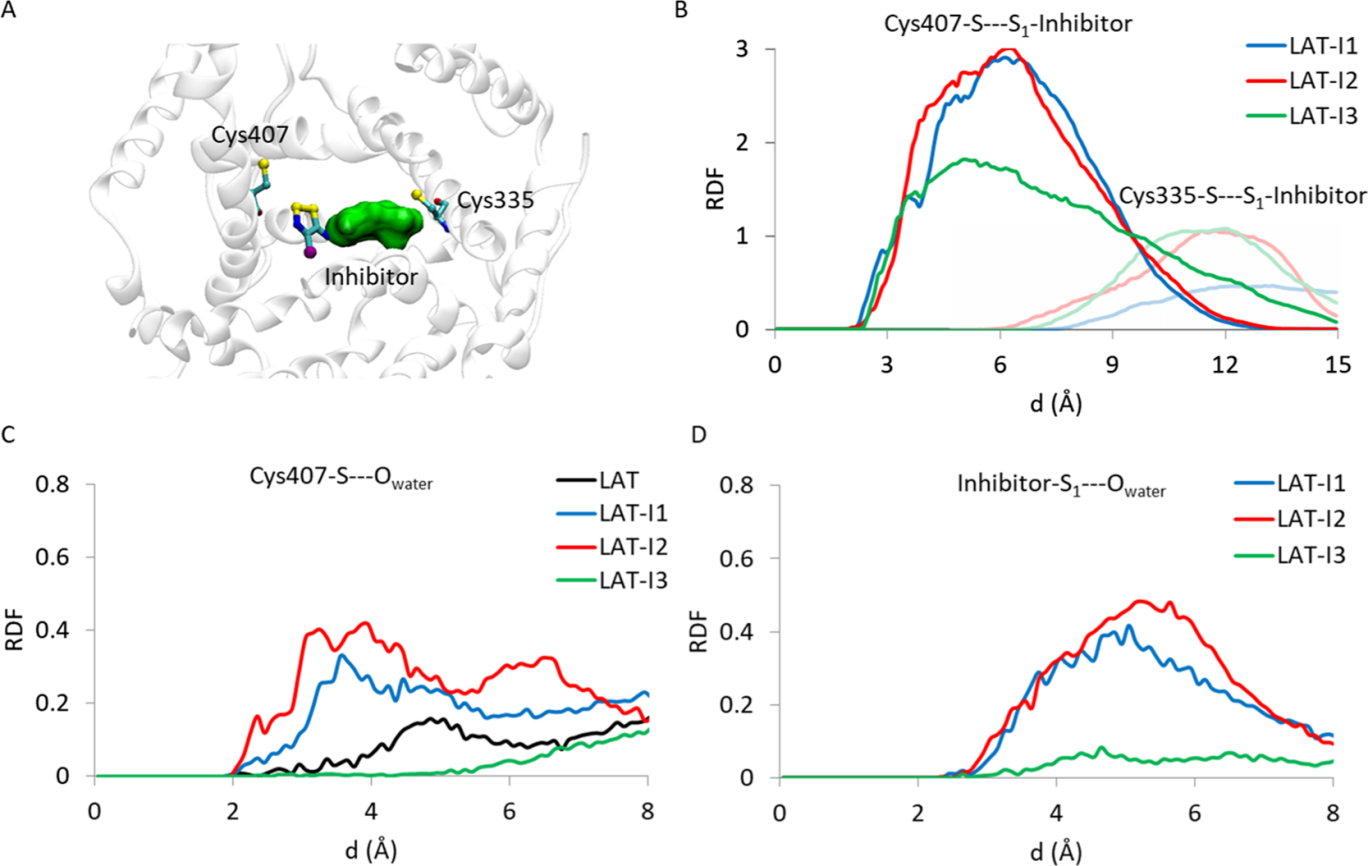
(A) Binding mode of the dithiazole scaffold into LAT1 binding cavity. Cys335 and Cys407 are depicted as cyan carbon sticks, the dithiazole group of the inhibitor able to form covalent bind to Cys407 is reported as cyan carbon sticks, and the other part of the inhibitor is displayed as the surface. LAT1 is shown as white transparent cartoon. Variation of distance between **I1**, **I2**, and **I3** and the sulfur atom of Cys335 and Cys407 (B), oxygen of water molecules, and sulfur atom of Cys407 (C) and **I1**, **I2**, and **I3** (D) during MDs by RDF.

The LAT1 binding pocket is characterized by hydrophobic residues such as Ile139, Val148, Phe252, Phe402, and Trp405, which ensure the strong interactions with the inhibitor. Polarizable residues, instead, such as Ser342, are engaged in hydrogen bond networks with different water molecules in all the MD simulation times. In particular, the calculation of radial distribution function (RDF) of the pair S_Cys407_–O_water_ during the MDs evidenced high probability of finding water molecules in proximity to Cys407 as confirmed by a broad peak in the range of 4.00 and 5.00 Å (see Figure 3A).

A similar behavior was also observed in cMD performed on the model of *apo-form* LAT1 starting from the cryoEM solved structure (see Supporting Information for details).^36^

From the MD trajectory cluster analysis,^27,28^ 10 representative structures of *apo-form* protein have been selected and subjected to the molecular recognition of three 1,2,3-dithiazoles(**I1**, **I2**, and **I3**) into the binding pocket of LAT1 proteins. The three best docked poses for each inhibitor complexed to LAT1 have been chosen for the next 100 ns MDs (Figure S3).

They were selected by applying the geometrical filter, such as the distance between the sulfur atom of the inhibitor and that of the Cys407 residue to facilitate the disulfide bond formation^56^ and according to the stronger theoretical binding affinity values (Table S1).

The results of MDs of the LAT1-inhibitor have also been investigated in terms of stability of the complexes and conformational flexibility of LAT1 in the presence of the selected inhibitors in comparison to the *apo-form*. RMSD trends of Cα, C, and N atoms for all complexes with respect to the initial structure have been reported, as shown in Figure S4A. In the presence of all the three inhibitors, as expected, an increased conformational stability of LAT1 in comparison to the unbound form (average RMSD_unboundLAT1_ value 3.68 Å) (see Figure S2) is observed. The average RMSD values for **I1**, **I2**, and **I3** in complex with LAT1 are 2.71, 2.47, and 3.19 Å, respectively.

The RMSF analysis (Figure S3A) reveals that protein regions containing Phe252, Cys335, Ser342, and Cys407 residues are characterized by small values, whereas pronounced fluctuations are observed in some loop regions.

The enhanced stability of the abovementioned residues can be related to their role in the accommodation/recognition of the inhibitors into the binding pocket.

The results, displayed in Figure S5, which evidence LAT1-**I1**, Ser66, and Phe252 residues, are associated to a decreased flexibility with respect to that of the unbound protein. Concerning LAT1-**I2**, the most significant shifts involve the Glu136, Phe252, Ser342, Trp405, and Ala409 residues, while in all cases, the Asn258 residue preserves a major stability in the presence of the ligands. Finally, we observe that for LAT1-**I3** with respect to the LAT1 complex with **I1** and **I2** inhibitors, the minor structural fluctuation observed is due to interaction with Trp405.

As shown in Figure S4B, the average distance between the sulfur atom of Cys407 and that of dithiazole moiety of **I1**, **I2**, and **I3** inhibitors was 4.51 Å, 4.84 Å, and 5.53 Å, respectively, meaning that these ligands can stably bind at the binding site.

To rationalize the different involvement of Cys335 and Cys407 in the binding site, the RDF has been evaluated for the three ligands and the S atom of the Cys335 and Cys407 residues (Figure 3B). Results show that, in all the three inhibitors, a minor distance between the inhibitor and Cys407 takes place. In addition, for **I1** and **I2**, the probability to form more stable interactions with Cys407 with respect to **I3** results to be higher. These structural insights evidence that Cys407 with respect to Cys335 represents the most favored target for the formation of the covalent protein-inhibitor complex, in agreement with the experimental findings.^12^ Both inhibitors and Cys407 displace proximal water molecules, which fill the LAT1’s channel during the MDs. In the case of amino acid residues, the RDF of the pair S_Cys407_–O_water_ (see Figure 3C) shows different peaks in the range of 2.00–4.55 Å for **I1** and **I2**, remarkably more intense than those calculated for the *apo-form* LAT1. The observed behavior is in line with the very recently cryoEM-solved LAT1 protein in complex with antigen 4F2hc and JX-078 inhibitor (PDB 7DSL), which displays one water molecule at 4.48 Å from S_Cys407_.^36^

A complementary behavior has been observed by examining the RDF of the pair S_inihibitor_–O_water_, which showed two intense peaks at 5.15 and 5.25 Å for **I1** and **I2** species, respectively. On the other hand, RDF for **I3** did not provide remarkable results. The fact that, in the course of the MD simulations, **I3** did not engage interactions with neither water molecules nor Cys407, the principal amino acid involved in the covalent inhibition, is in line with the weaker affinity measured of the species.^12^

On the other hand, in the case of species **I1** and **I2**, the more productive interactions between the inhibitor, Cys407, and water molecules can be crucial in the inhibition mechanism.

### Mechanistic insights into the role of the water molecules in the inhibition process by I1 and I2 from quantum chemical calculations

As mentioned above, the next step of the present investigation consisted of the investigation of the inhibition process based on the S–S covalent bond formation. Owing to the different behavior toward water molecules observed during the cMD investigations of the three inhibitor–protein complexes, the role of water molecules in the inhibition of LAT1 by **I1** and **I2** has been deeply examined and assessed.

In accordance to the recent proposal,^12^ the proposed reaction mechanism can proceed through two possible pathways (A and B).

**Scheme 3:**
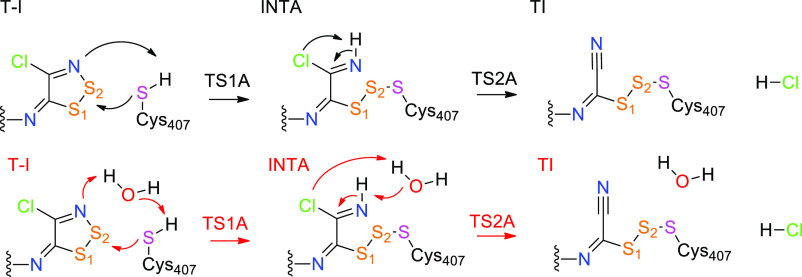
Proposed Reaction Mechanism for the Inhibition of LAT1 Occurring *via* the Trisulfide Intermediate (Mechanism A) (on Top) and the Respective Water-Mediated One (on Bottom)

Upon formation of the transporter protein-inhibitor (**T-I**) complex, in mechanism A, the nucleophilic attack of S_Cys407_ on the S2 of the dithiazole ring of the inhibitor takes place (**TS1A**), with asynchronous protonation of the N atom of the inhibitor, the opening of the thiazole ring, and formation of trisulfide species in **INTA**. The reaction is concluded by the elimination of Cl^–^ (**TS2A**) with the formation of the cyanic group (**TIA**) as experimentally observed (see Scheme 3).

In mechanism B, instead, the nucleophilic attack of S_Cys407_ takes place on S1 (**TSB**) and leads to a disulfide intermediate (**TIB**). In the proposed mechanisms, one water molecule can come into play acting as a proton shuttle in the transition states (see Figure S6).

Preliminary calculations on smaller protein-free models including Cys407, the inhibitor, and one water molecule (shown in Figures S7 and S8) have also been carried out. These computations and those concerning the larger model (see Figure 1) resulted in an energetic profile not suitable for the inhibition mechanism through disulfide bond formation (path B), with thermodynamically unfavorable formation of the covalent bond. This led us to discard this proposal and to mainly focus on mechanism A. All the detailed results concerning the mechanism B and the protein-free model are reported in the Supporting Information.

**Figure 4:**
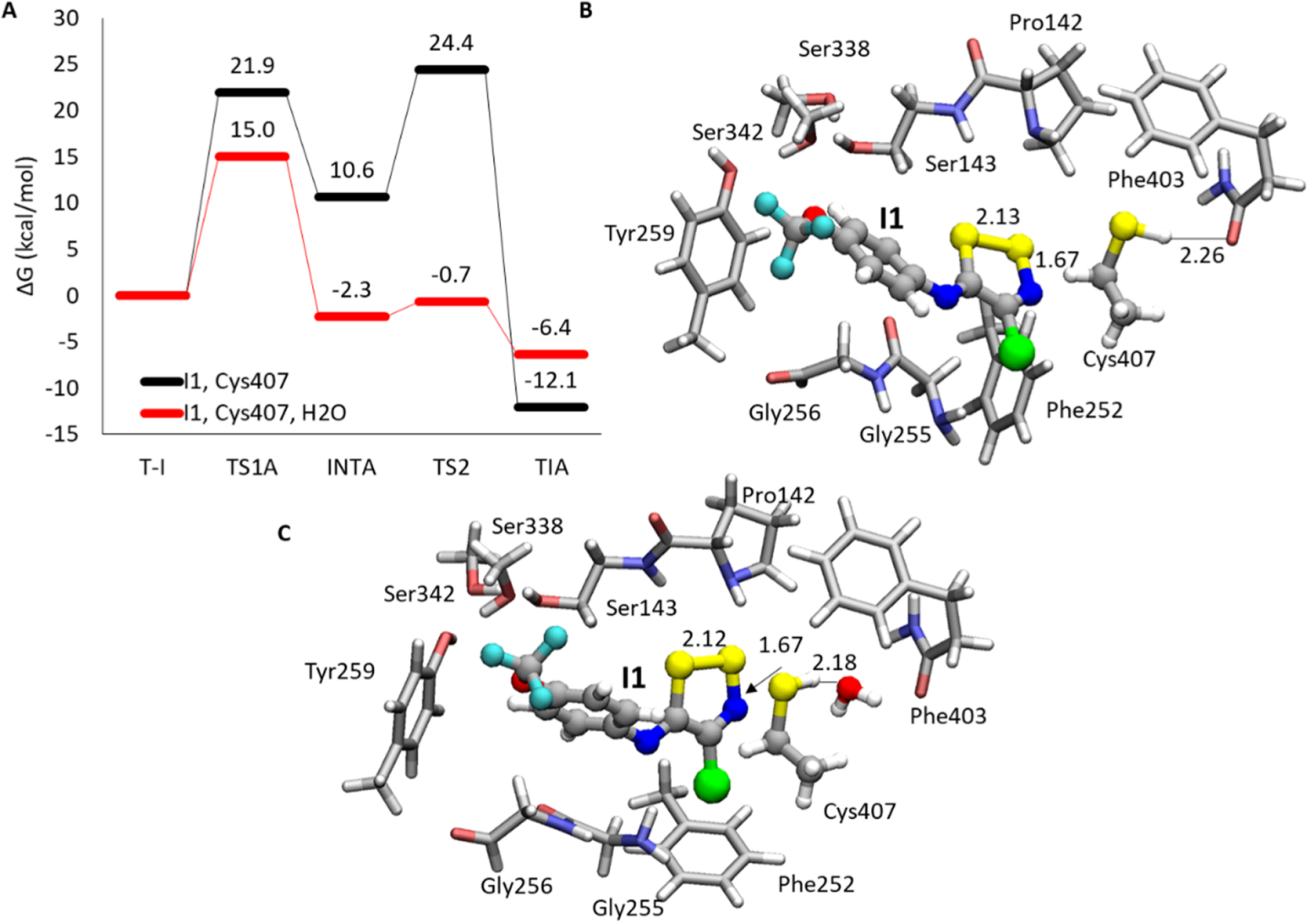
(A) ε = 4/B3LYP-D3/6-311+G(2d,2p):B3LYP-D3/6-31+G(d,p) relative energy surfaces calculated for inhibition of LAT1 by **I1**. B3LYP-D3/6-31+G(d,p) optimized structures of **T-I** (B) and **T-I-H**_**2**_**O** (C). Main distances are in Å.

The energy profiles calculated for the **I1** are reported in Figure 4A. Figure 4B,C shows water-free and water-containing optimized **T-I** complexes.

**Figure 5:**
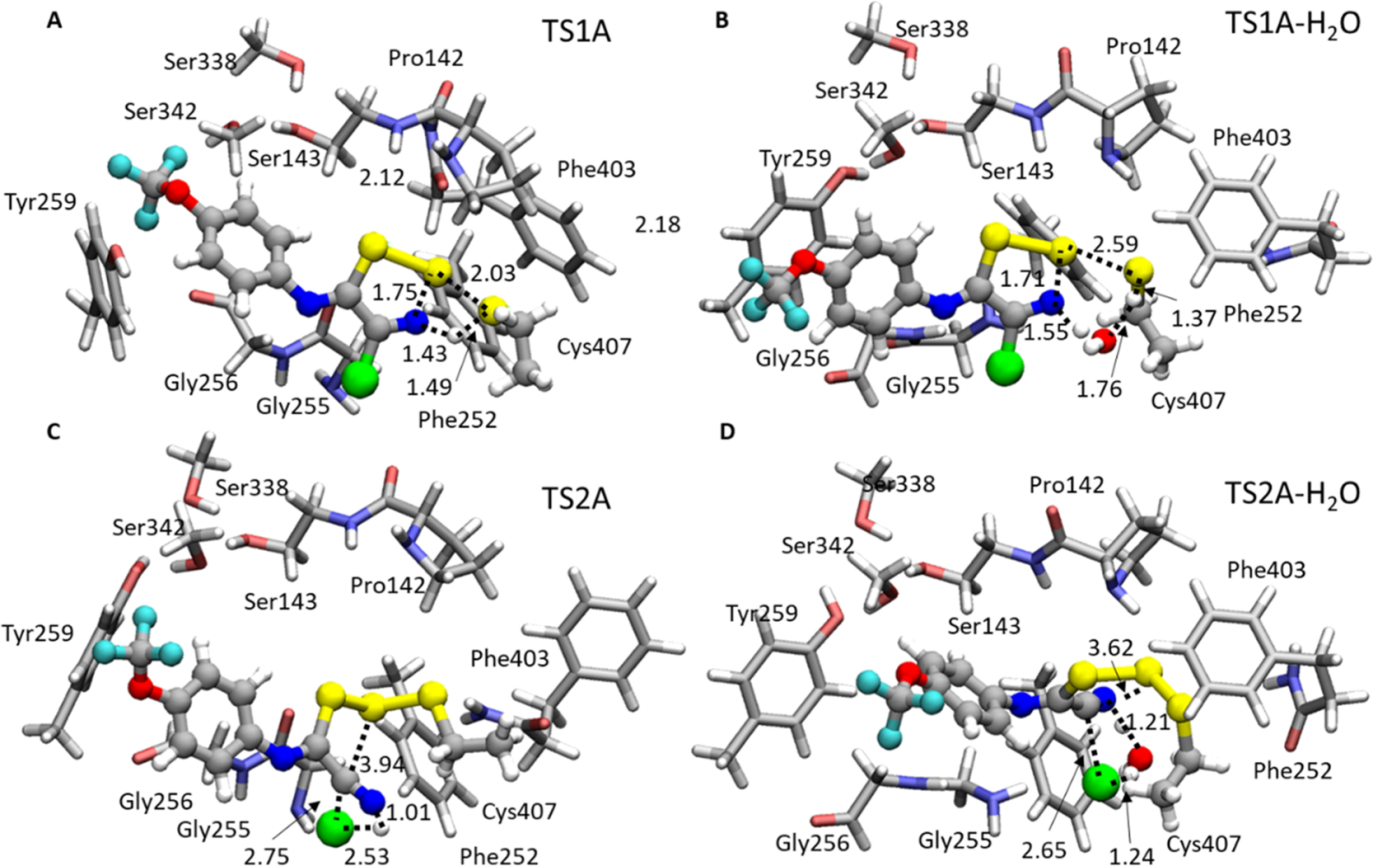
B3LYP-D3/6-31+G(d,p) optimized structures of **TS1A** (top) and **TS2A** (bottom) in the case of inhibition promoted by **I1**, in the absence (A,C) and presence of **H**_**2**_**O** (B,D). Main distances are in Å.

In the absence of water molecules, S_Cys407_ lies in a suitable way from S1 (4.01 Å) in **T-I** (see Figure 5). The distance, in the **TS1A** (see Figure 5), S_Cys407_–S1_**I1**_, decreases at 2.30 Å while the proton of thiol moiety is transferred to the N atom of the thiazole ring (1.43 Å). This process requires an energy barrier of 21.9 kcal/mol with respect to the reactant. The related intermediate **INTA** (+10.6 kcal/mol) presents the trisulfide bond formed, and a S1–N more elongated distance (1.79 vs 1.66 Å in **T-I**). The reaction proceeds toward products by overcoming the barrier represented by the **TS2A** of 24.4 kcal/mol energy above **T-I**. Here, the S1–N bond is cleaved (3.94 Å), and subsequently, Cl^–^ is released, as highlighted by the distance of 2.75 Å (1.73 Å in **INTA**) (see Figure 5). Due to the comparable values of **TS1A** and **TS2A** barriers, it is not possible to discriminate the rate-determining state of the reaction. The **TIA** final product (see Figure S8), lastly, is thermodynamically favored (−12.1 kcal/mol) with a reverse barrier equal to 36.5 kcal/mol typical of an irreversible inhibition process.

The water molecule explicitly included in the course of **T-I** optimization results to be involved in hydrogen bond interaction with thiol moiety of Cys407 (2.18 Å). In **TS1A**, water acts as proton shuttle donating hydrogen to N of the dithiazole ring (1.55 Å) and accepting hydrogen from S_Cys407_ (1.76 Å), thus favoring the formation of the S_Cys407_–S1 bond, as evidenced by the distance of 2.59 Å. This six-membered ring transition state has an energy of 15.0 kcal/mol, with respect to the reactant, lower by about 7 kcal mol than **TS1A** without water. This barrier represents the rate-limiting step of the mechanism. Regarding the reaction without water, the following **INTA** intermediate retains a transition state-like conformation, for the presence of the unformed S1–N bond now 3.13 versus 1.79 Å in the absence of water, caused by the interaction of NH with the water molecule (2.06 Å). In addition, the **INTA** lies at 2.3 kcal/mol below the reactant. The next **TS2A** lies at 1.6 kcal/mol above the previous minimum. During the transition state, the C–Cl distance increases to the value of 2.61 Å, from that present **INTA** (1.85 Å). In addition, analogously to **TS1A**, the water molecule donates one proton to Cl (1.93 Å) after accepting it from the N (1.24 Å) of **I1**. Also, in this case, the **TIA** shows favorable thermodynamics (−6.4 kcal/mol) for an irreversible inhibition.

**Figure 6:**
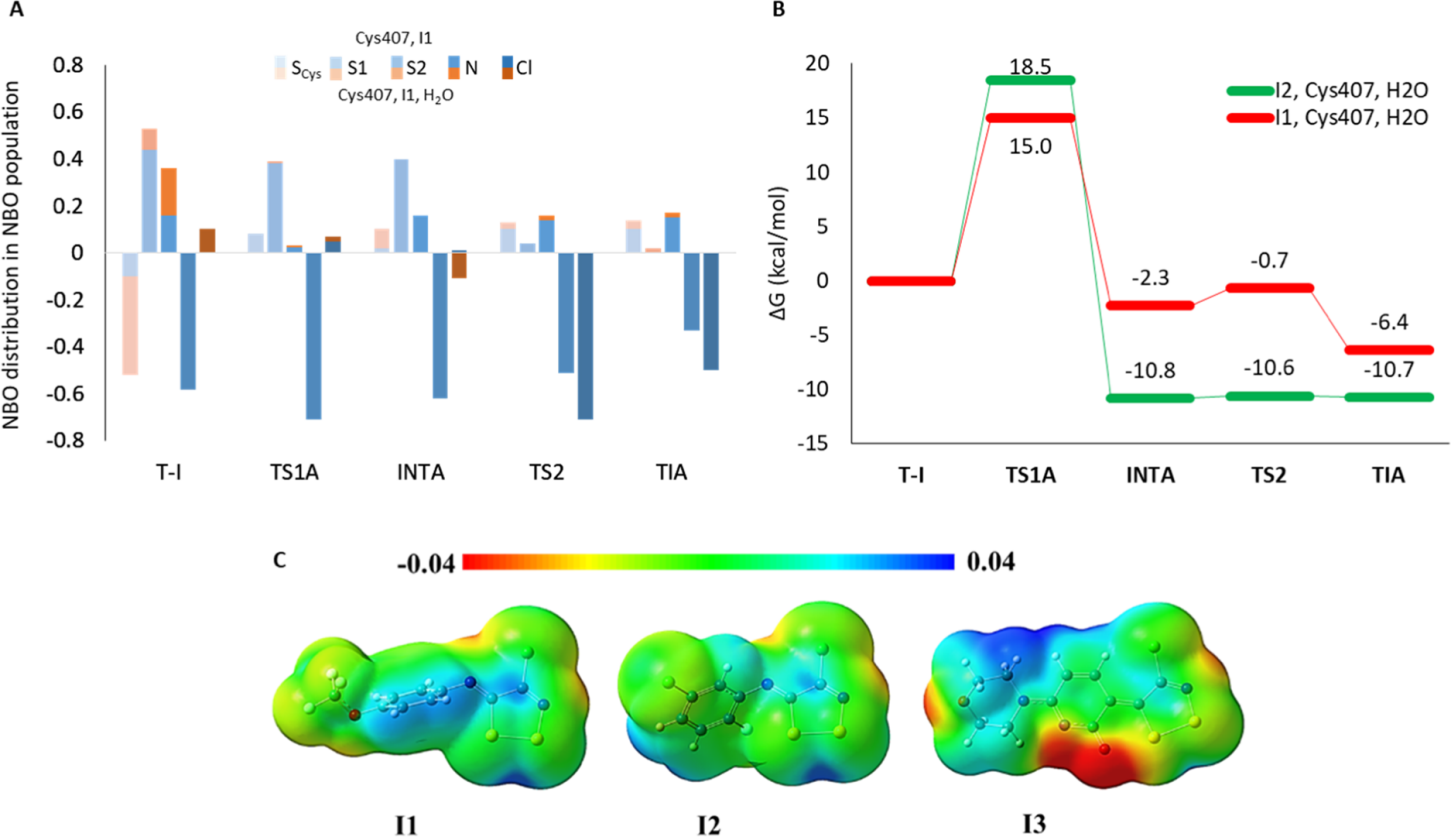
(A) NBO population analysis of selected atoms involved in the reaction mechanism. (B) ε = 4/B3LYP-D3/6-311+G(2d,2p):B3LYP-D3/6-31+G(d,p) relative energy surfaces calculated for inhibition of LAT1 by **I1** and **I2**. (C) Calculated MEPs for **I1**, **I2**, and **I3** species.

The analysis of energy profiles evidences the role played by the water molecule during the inhibition reaction. In particular, the most important contribution is observed in the stabilization of both six-membered ring transition states **TS1A** and **TS2A** (strained four-membered ring in the absence of water) and intermediate (**INTA**), as observed in a recent similar study.^15^ In the case of the process mediated by the water molecule, the **TS1A**, indeed, has 6.9 kcal/mol lower energy than the respective without water (15.0 vs 21.9 kcal/mol, respectively). In addition, the hydrogen bond engaging Cys407 and H_2_O enhances the nucleophile nature of S_Cys407_, as evidenced by the more negative charge resulted from the NBO analysis (|*e*| = −0.5 with H_2_O vs |*e*| = −0.1 without H_2_O, Figure 6A). However, the calculated barriers for both inhibition processes (with and without H_2_O, Figure 4A) well fit the available theoretical values for the formation of the S–S bond (∼20 kcal/mol).^57,58^

Both **INTA** and **TS2A** are stabilized in the energy of 12.9 and 25.1 kcal/mol, respectively, in comparison with the corresponding ones in the absence of H_2_O, mainly by the hydrogen bond interactions involving the inhibitor and the water molecule. In particular, the **INTA** shows a **TS2A**-like conformation, with elongation of S1–N and Cl–C bonds favored by the presence of hydrogen bonds with H_2_O. This makes “unstable” the intermediate, and consequently, the inhibition can more easily proceed. This aspect is further evidenced by the analysis of NBO charges calculated for the stationary point. In the case of the Cl atom, the presence of the water molecule induced the localization of more negative charges in the **INTA**, analogous to the charge calculated in the following **TS2A** (see Figure 6A), thus explaining the difference in the energy calculated for the relative **INTA**-**TS2A** barrier (1.4 vs 13.8 kcal/mol in the presence and absence of water, respectively).

It should be mentioned that in the case of water-mediated inhibition mechanism, the kinetic of reversible reaction (**TI** → **T-I**) is less favored. Indeed, for this reaction, an energy barrier of 21.4 kcal/mol must be overcome, resulting 6.4 kcal/mol higher than that obtained for the **T-I** → **TI** path. The highlighted trend well matches the observed irreversible inhibition measured in the presence of **I1** species.^12^

Once established the role of water molecules in the inhibition mechanism of LAT1 by **I1**, the reaction has been investigated also for **I2** species, which presents IC_50_ values in proximity with the former inhibitor.^12^ The potential energy surfaces are shown in Figure 6B, while all stationary points intercepted along the inhibition mechanism are provided in the Supporting Information.

**I2** presents an energy barrier of 18.5 kcal/mol, in proximity with **TS1A**, 3.5 kcal/mol higher with respect to **I1**. After that, the reaction proceeds fast below the reactant (Δ*G*_INTA-TS2A_ = 0.2 kcal/mol), with favorable thermodynamics, as evidenced by the energy of −10.7 kcal/mol of **TIA**.

In general, the analysis of **I1** and **I2** energy profiles and optimized geometries (see Supporting Information) highlights similar activity of the two inhibitors, in line with the IC_50_ measured for the two species.^12^ However, the inhibitors have a different electrostatic nature, which was investigated by plotting molecular electrostatic potential (MEP). **I1**, **I2**, and **I3** (see Figure 6C) evidenced electrophilicity of the dithiazol ring, in the part of the molecule that undergoes to the nucleophilic attack by Cys407. Indeed, despite in proximity with the S1–S2 bond, there are no appreciable differences between the two species, the **I1’s** dithiazole ring presents a more positive blue region with respect to that one localized for **I2** (cyan), indicating higher electrophilicity of the molecule, which could explain the slightly different stability of **TS1A** (see Figure 6B).

**I3**’s green area is characterized by the lowest electrophilicity, which well correlates with the weak affinity for LAT1.^12^ Finally, the richer electron region localized on the C–Cl bond of **I2**, with respect to **I1**, can indicate the higher predisposition as leaving group by the Cl group, in **TS2A**, thus explaining the difference in behavior determined as shown in Figure 6B.

## Conclusions

In the present computational study, we have investigated the inhibition mechanism of the LAT1 transporter by 1,2,3 dithiazoles derivatives.

Four different MDs for apo-LAT1 and the **I1-**, **I2-**, and **I3-**bound proteins have been performed, after molecular docking of the inhibitors, for the initial all-atoms MD study. The simulations performed on apo-LAT1 highlighted that Ser66, Glu136, Asn258, Ser342, Trp405, and Ala409, located in the highly conserved region TMs TM1, TM3, TM6, TM8, and TM10, are the residues mainly involved in the substrate recognition.

The binding site for the inhibitor is, instead, composed of Pro142, Ser143, Ile195, Gly256, Cys335, Asn404, Cys407, and Val408. The analysis of the trajectories carried out through the study of rmsd, RMSF and RDF showed that both **I1** and **I2** inhibitors engage stable interactions with Cys407, the nucleophile agent in the inhibition mechanism, more than **I3**. Intriguingly, the MDs of the inhibitor-bound protein highlighted the frequent presence of water molecules in proximity with Cys407 and **I1** and **I2** molecules. Both behaviors can be related to the higher activity of these species with respect to **I3**, thus explaining experimental outcomes.^12^

In addition, the inhibition mechanism of LAT1 by **I1** has been initially investigated in the mean of DFT, taking further into account the effect of H_2_O. The outcomes from QM calculations propose, among the reaction mechanisms,^12^ the reaction proceed through formation of the trisulfide bond between Cys407 and the inhibitor (mechanism A) with an energetic amount of +21.9 kcal/mol, corresponding to the attack of S_Cys407_ to the S2 of the inhibitor (mechanism B).

In the presence of one water molecule, an important stabilization of the transition states (from +21.9 to +15.0 kcal/mol, for the rate-determining step) and the intermediates of the reaction takes places. This is mainly corroborated by the presence of the hydrogen bond network. These results, in addition to those of the MDs, confirm the important role played by the water molecule in the inhibition.

Finally, the comparative study between **I1** and **I2** provides explanation for the experimentally observed activity of the inhibitors.

We hope that the results presented here on the inhibition of LAT1 can be useful in the rational design of new and more potent inhibitors.

## Data and Software Availability

The preliminary docking study was done adopting AutoDock version 4.2. MD simulations were performed adopting the AMBER16 package. AmberTools package was further adopted in the preparation of parameters and input for MD simulations. DFT calculations were carried out using Gaussian 09 ver. D01. The preparation of the structure and the analysis of trajectories and the images were carried out using visual MD software, version 1.9.3. The full workflow is reported in the “Computational Methods” section of the manuscript.

## Supporting Information Available

Additional results of MD simulations performed on apo-LAT1 and **I1**-, **I2**-, and **I3**-LAT1 systems; results on the alternative B pathway; images of optimized species involved; and list of parameters in the AMBER16 format (PDF)
MD of **I1**-bound LAT1 (MP4)
