# Supplementary material for: Computational Study Reveals the Role of Water Molecules in the Inhibition Mechanism of LAT1 by 1,2,3-Dithiazoles
Source: J Chem Inf Model. 2021 Nov 17;61(12):5883–92. doi: 10.1021/acs.jcim.1c01012 (PMC8715508; doi:10.1021/acs.jcim.1c01012)
Supplement: Supplementary file 1 — ci1c01012_si_001.pdf [file ci1c01012_si_001.pdf]

Supporting Information

Computational study reveals the role of water molecules in the inhibition mechanism of LAT1 by 1,2,3-dithiazoles

Mario Prejanò, Isabella Romeo, Maria Antonietta La Serra, Nino Russo, Tiziana Marino

Department of Chemistry and Chemical Technologies, University of Calabria, 87036 Arcavacata di Rende, Cosenza, Italy

* Correspondence should be addressed to

tiziana.marino65@unical.it

mario.prejano@unical.it

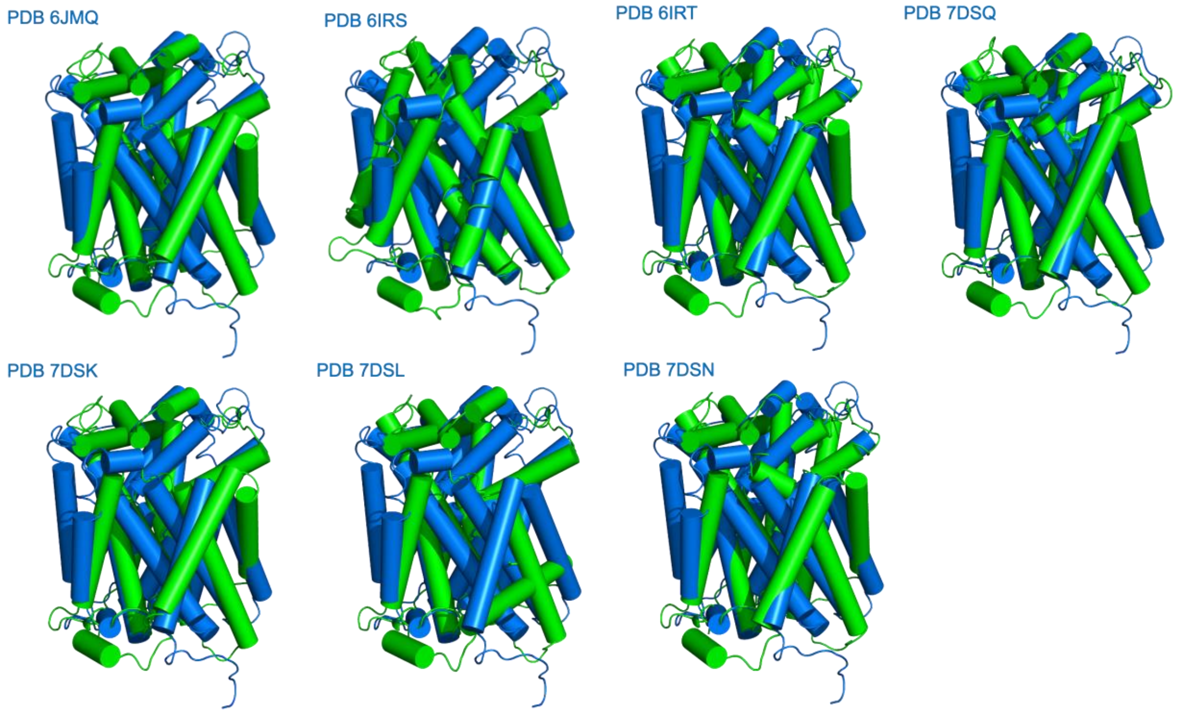

Figure S1. Superpositions of LAT1 protein obtained from homology modeling structure (green) and different cryoEM solved structures (blue).

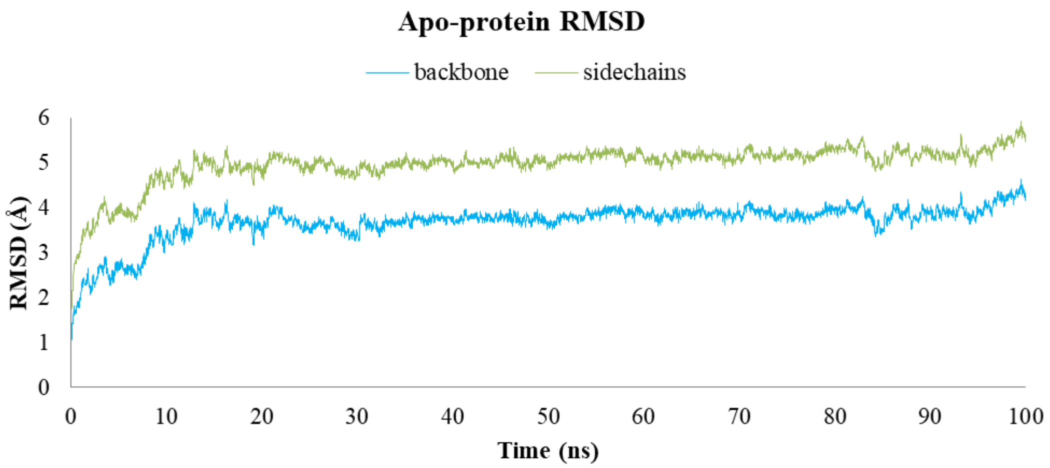

Figure S2. RMSD plot of LAT-1 protein backbone (blue line) and side-chains atoms (green lines).

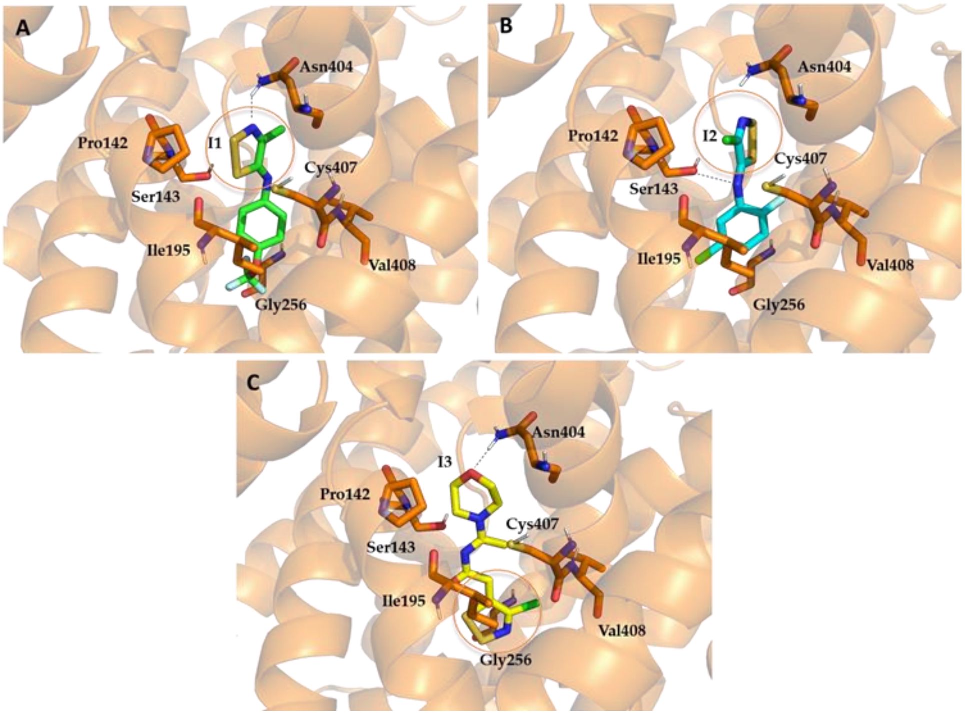

Figure S3. In panels A), B) and C) binding mode of I1, I2 and I3 against LAT1 are reported, respectively. LAT1 structure and residues involved in crucial contacts with the inhibitors are depicted as orange cartoon and carbon sticks, respectively. I1, I2 and I3 are represented as green, cyan and yellow carbon sticks. The hydrogen bonds between the inhibitor and LAT1 are shown as dashed black lines and the dithiazole moiety is surrounded by the orange circle.

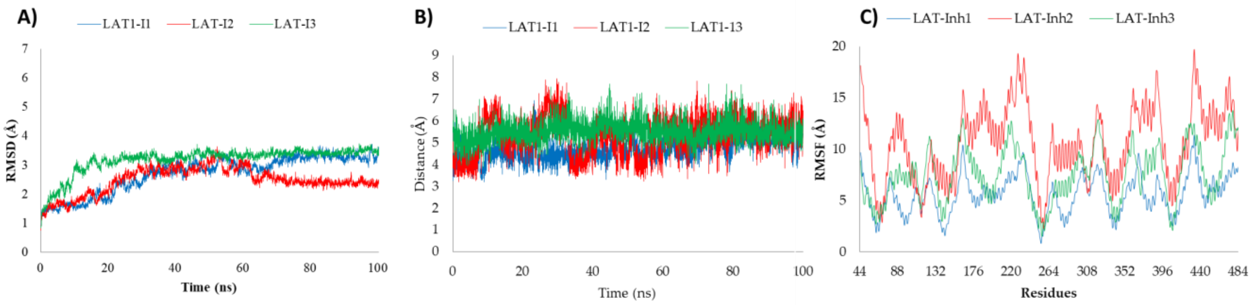

Figure S4. Panel A) RMSD trend, B) distance between S_{Cys407} and $S_{Inhibitor}$, C) RMSF of every residue in LAT1in complex to I1, I2 and I3 during 100 ns of MDs, reported in blue, red and green lines, respectively.

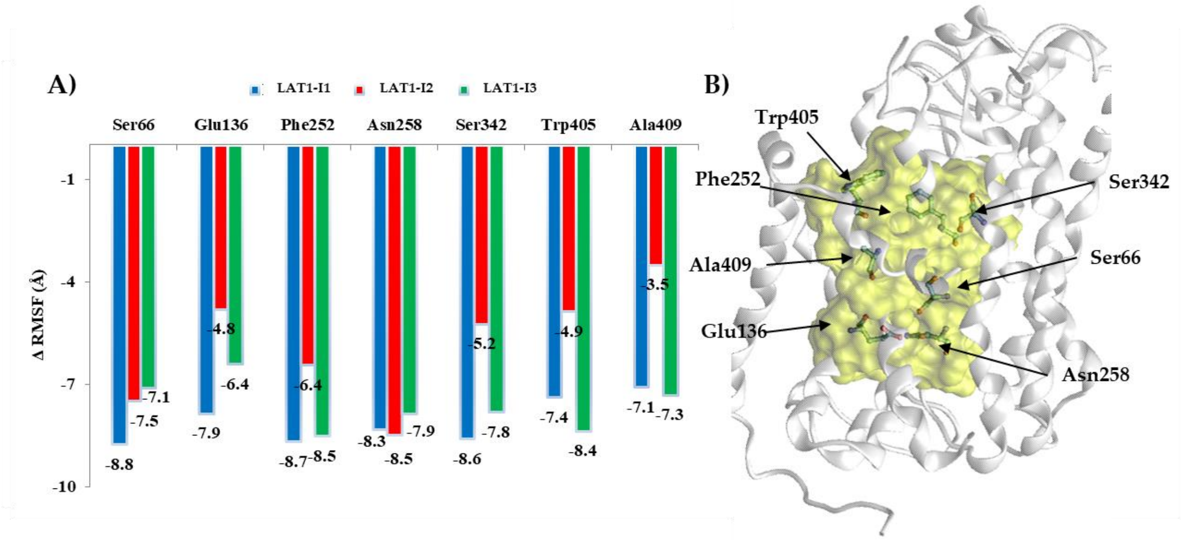

Figure S5. Difference (Δ) between the RMSF value (Å) of these residues extracted by MDs of the three ligands in complex to LAT1 and that of apo-form protein MDs. ($\Delta RMSF = RMSF_{boundprotein} - RMSF_{unboundprotein}$.

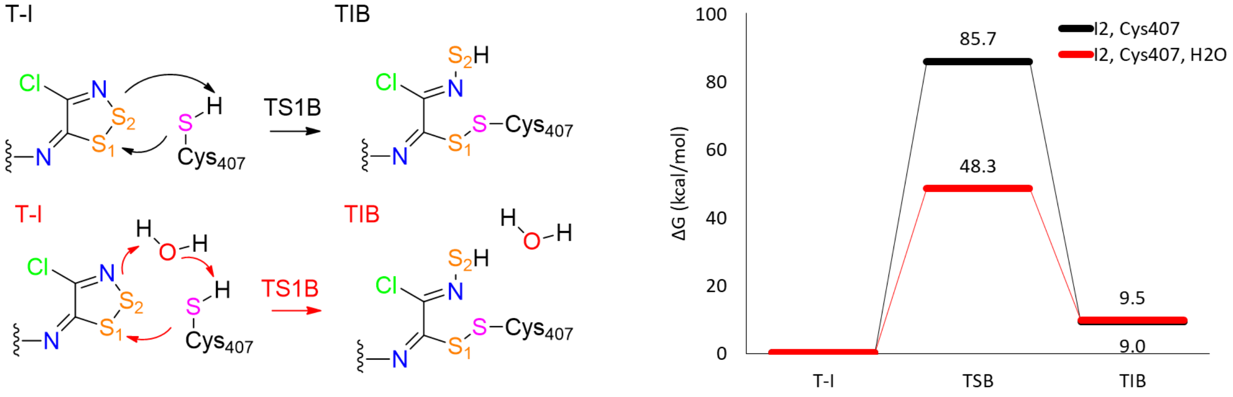

Figure S6. On the left, The proposed reaction mechanism for the inhibition of LAT1 occurring via disulphide intermediate (mechanism B), on top, and the respective water-mediated one (on bottom) and, on the right, the related ε=4/B3LYP-D3/6-311+G(2d,2p):B3LYP-D3/6-31+G(d,p) relative energy surfaces calculated for inhibition of LAT1 by I1.

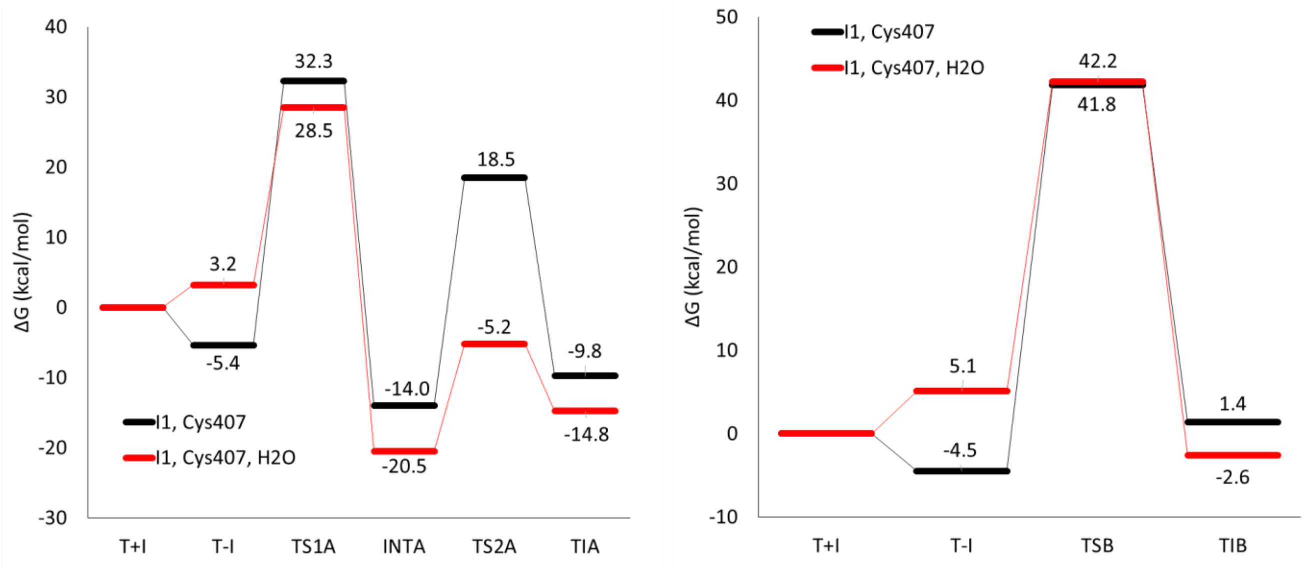

Figure S7. ε=4/B3LYP-D3/6-311+G(2d,2p):B3LYP-D3/6-31+G(d,p) relative energy surfaces calculated for inhibition of LAT1 by I1 *via* mechanism A (left) and B (right), for the protein-free model.

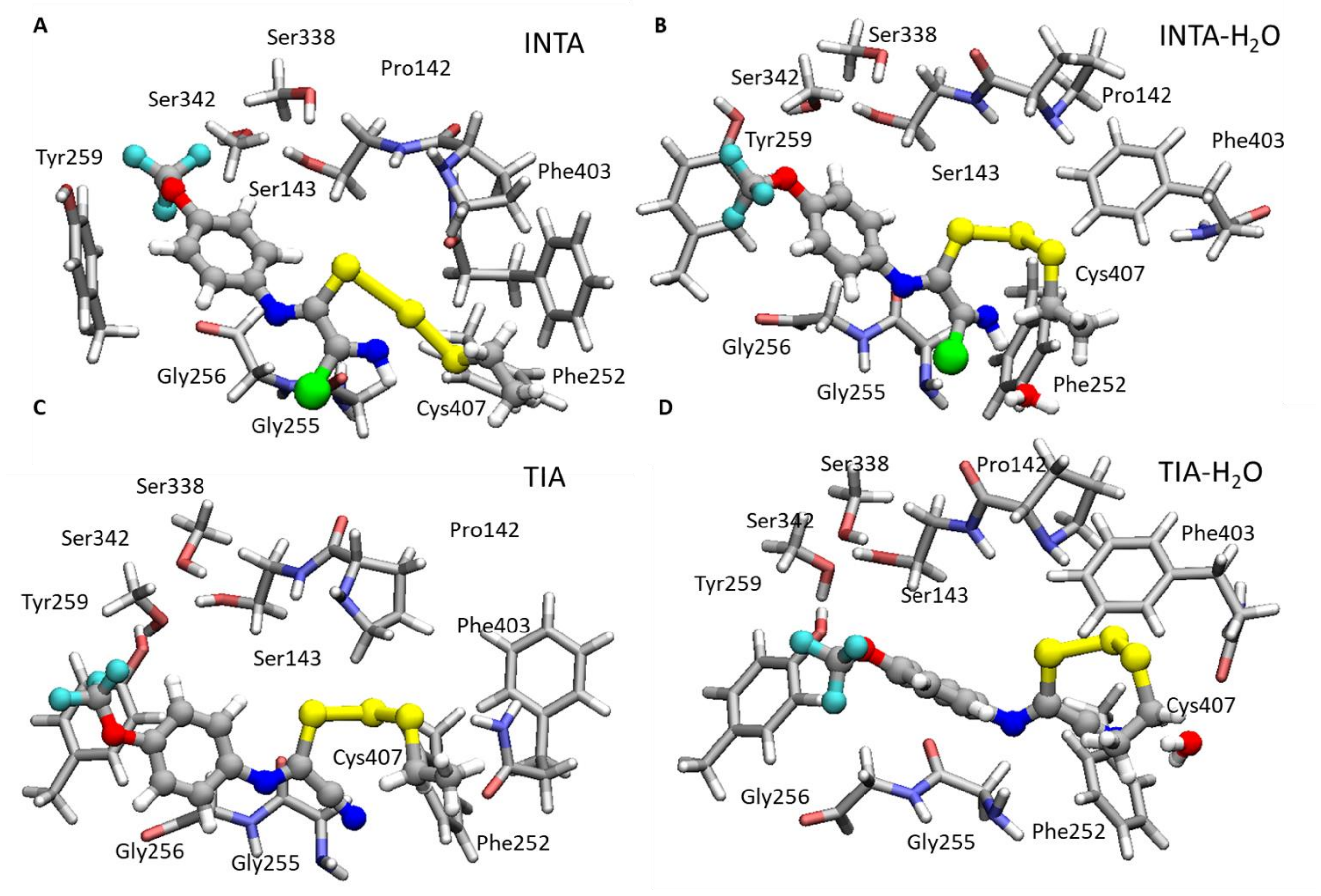

Figure S8. B3LYP-D3/6-31+G(d,p) optimized structures of INTA (top) and TIA (bottom) in the case of inhibition promoted by I1, in absence (left) and presence of H_2 O (right).

Table S1. Calculated binding energies (E_b) for different docking poses, in kcal/mol. In parenthesis, the S_{Cys} - $S_{Inhibitor}$ is reported in Å.

	E_b* (S_{Cys} - $S_{Inhibitor}$ atomic distance)		
	I1	I2	I3
Cluster 1	-6.32 (3.21)	-6.39 (5.57)	-6.22 (6.37)
Cluster 2	-5.52 (6.71)	-6.00 (6.67)	-5.29 (9.17)
Cluster 3	-5.70 (5.73)	-5.98 (8.46)	-6.15 (8.87)
Cluster 4	-6.31 (7.30)	-5.36 (7.64)	-6.12 (9.90)
Cluster 5	-5.80 (7.98)	-5.98 (8.15)	-6.01 (8.53)
Cluster 6	-5.00 (6.80)	-5.51 (7.99)	-5.97 (8.25)
Cluster 7	-6.35 (6.78)	-5.51 (7.98)	-5.94 (9.66)
Cluster 8	-5.39 (8.73)	-5.42 (9.80)	-5.92 (8.25)
Cluster 9	-5.21 (6.50)	-5.51 (6.91)	-5.80 (8.19)
Cluster 10	-5.90 (7.34)	-5.31 (8.47)	-5.78 (9.36)

Table S2. Calculated pKa for ionizable residues. Residues highlighted in blue or red are those lying in positive, fully protonated, or negative, fully de-protonated state respectively.

Residue	Calculated pKa	Residue	Calculated pKa
THR-1	7.167	GLU-221	<0.000
ARG-4	>12.000	GLU-222	1.525
LYS-33	>12.000	TYR-227	10.2
GLU-34	1.264	ARG-228	11.439
CYS-48	>12.000	TYR-245	>12.000
CYS-58	>12.000	TYR-252	>12.000
TYR-59	>12.000	GLU-259	4.267
GLU-61	<0.000	GLU-265	4.12
LYS-68	>12.000	ASP-270	2.406
ASP-72	<0.000	TYR-274	11.389
TYR-73	>12.000	HIS-275	3.801
TYR-75	>12.000	CYS-291	>12.000
GLU-78	4.85	ARG-304	>12.000
TYR-80	8.939	ARG-311	>12.000
LYS-88	3.903	GLU-312	<0.000
GLU-92	5.29	HIS-314	3.725
ARG-97	10.344	HIS-323	4.73
TYR-102	>12.000	CYS-337	10.031
TYR-111	10.975	TYR-343	>12.000
LYS-114	>12.000	LYS-347	8.527
CYS-120	>12.000	ASP-348	2.916
GLU-124	4.467	CYS-363	>12.000
GLU-125	4.054	ARG-375	>12.000
LYS-128	10.787	HIS-376	<0.000
CYS-132	>12.000	ARG-377	>12.000
CYS-134	>12.000	LYS-378	8.861
CYS-143	11.23	GLU-380	3.355
TYR-144	11.041	GLU-382	2.814
LYS-147	9.007	ARG-383	>12.000
ARG-151	>12.000	LYS-386	9.992
ASP-154	4.002	CYS-407	11.177
LYS-160	8.693	LYS-409	>12.000
LYS-177	9.835	GLU-413	1.414
ASP-179	3.026	CYS-414	>12.000
ASP-184	3.966	TYR-428	>12.000
GLU-190	4.949	LYS-435	10.962
LYS-193	10.631	LYS-437	9.957
ASP-195	4.654	LYS-439	10.864
TYR-204	10.48	GLY-444	2.677
TYR-210	>12.000		
TYR-215	>12.000		

Additional Molecular Dynamics simulations on LAT1 starting from cryoEM structure

Molecular dynamics simulation was additionally performed on apo-form LAT1, starting from the very recently solved cryo electron-microscopy (cryoEM) structure of LAT1 in complex with the antigen heavy chain 4F2hc and the JX-078 inhibitor (PDB 7DSL), [1] in order to investigate the effect of different initial structure to the structural outcomes.

Following the same procedure described in the main text, the LAT1 protein was protonated and counter ions were added. A number of water molecules was explicitly retained in the model also. The system was fully solvated with TIP3P model for water, adding an orthorhombic box with a buffer of 10 Å. The ff14SB force field [2] has been adopted as implemented in AMBER16. [3] The system was firstly minimized and heated for 50 ps at 300 K. Finally, the production phase of 50 ns was performed. The minimizations and MD simulations were carried out in agreement to the procedure described in the Computational Methods section of manuscript.

Any relevant structural variations were not observed in the course of the simulations. The superposition of last frame of *c*MD simulation well fits the homology model (see **Figure S9A**). This result confirms the structural similarities that were observed between homology modeling of LAT1 as with PDB 7DSL as with other available cryoEM structures (**Figure S1**), in particular regarding the secondary structures composing the trans-membrane protein.

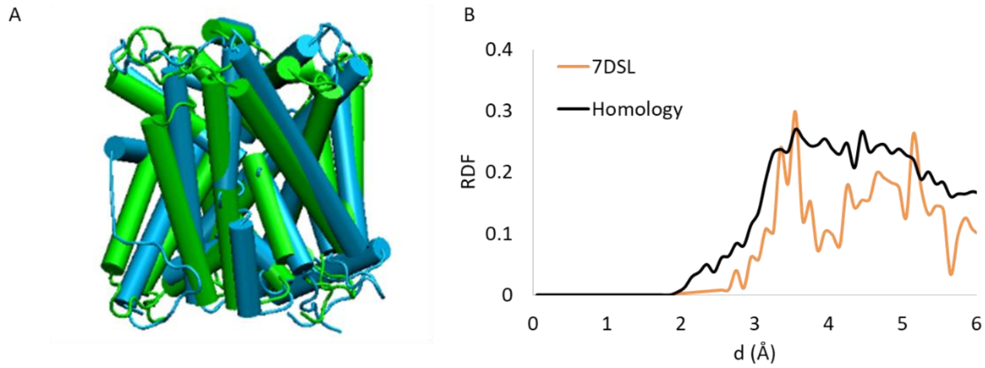

Figure S9. A) Superpositions of apo-form LAT1 protein from homology modeling (green) and the last frame of *c*MD performed starting from cryoEM solved structure (PDB 7DSL, in blue). B)

More interestingly, the comparison of the radial distribution function of S_{Cys407} -O_{Water} pair of the apo-form LAT1 from homology modeling and from cryoEM, in **Figure S9B**, highlights an analogous behavior of the water molecules. Indeed, line with the results discussed in the main manuscript, the presence of water molecules in proximity of thiol group of Cys407 is demonstrated by a number of peaks

in the range of 2.75 Å -5.85 Å (see **Figure S9B**). The most intense peaks were observed at 3.55 Å and 5.15 Å, corresponding to the first and second hydration shells of Cys407' thiol group.

References

[1] Yan, R.; Li, Y.; Müller, J.; Zhang, Y.; Singer, S.; Xia, L.; Zhong, X.; Gertsch, J.; Altmann, K.H.; Zhou, Q. *Cell Discov.* **2021**, **7,** 16.

[2] Maier, J.A.; Martinez, C.; Kasavajhala, K.; Wickstrom, L.; Hauser, K.E.; Simmerling, C. ff14SB: Improving the accuracy of protein side chain and backbone parameters from ff14SB. *J. Chem. Theory Comput.* **2015**, *11*, 3696–3713.

[3] Case, D.A.; Cheatham, T.E.; Darden, T.; Gohlke, H.; Luo, R.; Merz, K.M.; Onufriev, A.; Simmerling, C.; Wang, B.; Woods, R.J. *J. Comput. Chem.* **2005**, 26, 1668–1688.

List of parameters in Amber FF format

I1.prepc

```
remark goes here
MASS
ca 12.010       0.360
ha 1.008        0.135
ne 14.010       0.530
cd 12.010       0.360
nc 14.010       0.530
ss 32.060       2.900
cl 35.450       1.910
os 16.000       0.465
c3 12.010       0.878
f  19.000       0.320

BOND
ca-ca  461.10   1.398
ca-ne  389.30   1.408
ca-ha  345.80   1.086
ne-cd  560.30   1.298
cd-cd  419.80   1.428
cd-ss  265.80   1.756
cd-nc  525.40   1.317
cd-cl  317.50   1.735
nc-ss  359.80   1.626
ss-ss  153.80   2.073
ca-os  376.60   1.370
os-c3  308.60   1.432
c3-f   356.90   1.350

ANGLE
ca-ca-ca   66.620     120.020
ca-ca-ha   48.180     119.880
ca-ne-cd   65.630     123.670
ca-ca-ne   67.850     120.610
ca-ca-os   69.580     119.200
ne-cd-cd   69.220     121.680
ne-cd-ss   63.090     126.000
cd-cd-nc   71.570     112.560
cd-cd-cl   57.800     119.990
cd-ss-ss   63.530      93.800
cd-cd-ss   61.930     120.210
cd-nc-ss   68.550     108.070
nc-cd-cl   59.720     122.070
nc-ss-ss   65.090      97.440
ca-os-c3   62.520     117.960
os-c3-f    70.760     110.580
f -c3-f    70.890     107.360

DIHE
ca-ca-ca-ha   1    3.625      180.000       2.000
ca-ca-ca-ca   1    3.625      180.000       2.000
ca-ne-cd-cd   1    0.800      180.000       2.000     same as X -ce-ne-X
ca-ne-cd-ss   1    0.800      180.000       2.000     same as X -ce-ne-X
ca-ca-ne-cd   1    0.000      180.000       3.000
ca-ca-ca-os   1    3.625      180.000       2.000
ca-ca-ca-ne   1    3.625      180.000       2.000
ca-ca-os-c3   1    0.900      180.000       2.000
ha-ca-ca-ha   1    3.625      180.000       2.000
ha-ca-ca-os   1    3.625      180.000       2.000
ha-ca-ca-ne   1    3.625      180.000       2.000
ne-cd-cd-nc   1    4.000      180.000       2.000
ne-cd-cd-cl   1    4.000      180.000       2.000
ne-cd-ss-ss   1    1.100      180.000       2.000     same as X -c2-ss-X
cd-cd-nc-ss   1    4.750      180.000       2.000
cd-ss-ss-nc   1    0.000        0.000       3.000
cd-cd-ss-ss   1    1.100      180.000       2.000     same as X -c2-ss-X
cd-nc-ss-ss   1    4.800      180.000       2.000
nc-cd-cd-ss   1    4.000      180.000       2.000
ss-nc-cd-cl   1    4.750      180.000       2.000
ss-cd-cd-cl   1    4.000      180.000       2.000
ca-os-c3-f    1    0.383        0.000       3.000

IMPROPER
ca-ca-ca-ne      1.1      180.0      2.0      Using default value
ca-ca-ca-ha      1.1      180.0      2.0      General improper
torsional angle (2 general atom types)
cd-ne-cd-ss      1.1      180.0      2.0      Using default value
cd-cl-cd-nc      1.1      180.0      2.0      Using default value
ca-ca-ca-os      1.1      180.0      2.0      Using default value

NONBON
  ca        1.9080  0.0860
  ha        1.4590  0.0150
  ne        1.8240  0.1700
  cd        1.9080  0.0860
  nc        1.8240  0.1700
  ss        2.0000  0.2500
  cl        1.9480  0.2650
  os        1.6837  0.1700
  c3        1.9080  0.1094
  f         1.7500  0.0610
```

I1.prepc

```
   0    0    2

This is a remark line
molecule.res
MOL   XYZ  0
CHANGE     OMIT DU   BEG
  0.0000
   1  DUMM  DU    M      999.000     999.0      -999.0        .00000
   2  DUMM  DU    M      999.000    -999.0       999.0        .00000
   3  DUMM  DU    M     -999.000     999.0       999.0        .00000
   4  C1    ca    M    -0.024000   0.454000   0.071000   0.321745
   5  C3    ca    B     0.543000  -0.386000  -0.878000  -0.159878
   6  C5    ca    S     1.871000  -0.765000  -0.765000  -0.281122
   7  H4    ha    E     2.322000  -1.414000  -1.492000   0.202499
   8  H2    ha    E    -0.040000  -0.728000  -1.712000   0.162560
   9  N1    ne    S    -1.355000   0.902000  -0.032000  -0.540187
  10  C8    cd    S    -2.339000   0.145000  -0.017000   0.364571
  11  C9    cd    B    -3.741000   0.664000  -0.124000   0.337862
  12  N2    nc    S    -4.724000  -0.099000  -0.048000  -0.397782
  13  S2    ss    S    -4.436000  -1.731000   0.163000   0.171146
  14  S1    ss    E    -2.351000  -1.630000   0.168000  -0.113945
  15  Cl1   cl    E    -3.964000   2.352000  -0.341000  -0.072914
  16  C2    ca    M     0.750000   0.931000   1.122000  -0.159878
  17  H1    ha    E     0.310000   1.600000   1.837000   0.162560
  18  C4    ca    M     2.070000   0.541000   1.245000  -0.281122
  19  H3    ha    E     2.674000   0.888000   2.062000   0.202499
  20  C6    ca    M     2.618000  -0.301000   0.296000   0.324840
  21  O1    os    M     3.927000  -0.732000   0.446000  -0.374225
  22  C7    c3    M     4.906000  -0.017000  -0.113000   0.725420
  23  F2    f     E     4.951000   1.216000   0.350000  -0.198216
  24  F3    f     E     6.041000  -0.603000   0.157000  -0.198216
  25  F1    f     M     4.778000   0.063000  -1.422000  -0.198216


LOOP
   C6   C5
   S1   C8

IMPROPER
   C2   C3   C1   N1
   C1   C5   C3   H2
   C6   C3   C5   H4
   C9   N1   C8   S1
   C8   Cl1  C9   N2
   C4   C1   C2   H1
```

```
  C2   C6   C4   H3
  C4   C5   C6   O1

DONE
STOP
```

I2.frcmod

```
remark goes here
MASS
cc 12.010        0.360
nd 14.010        0.530
ss 32.060        2.900
cl 35.450        1.910
nf 14.010        0.530
ca 12.010        0.360
ha 1.008         0.135
f  19.000        0.320

BOND
cc-cc  419.80   1.428
cc-ss  265.80   1.756
cc-nf  560.30   1.298
cc-nd  525.40   1.317
cc-cl  317.50   1.735
nd-ss  359.80   1.626
ss-ss  153.80   2.073
nf-ca  389.30   1.408
ca-ca  461.10   1.398
ca-f   357.80   1.349
ca-ha  345.80   1.086
ca-cl  305.60   1.750

ANGLE
cc-cc-nd   71.570     112.560
cc-cc-cl   57.800     119.990
cc-ss-ss   63.530     93.800
cc-nf-ca   65.630     123.670
cc-cc-ss   61.930     120.210
cc-cc-nf   69.220     121.680
cc-nd-ss   68.550     108.070
nd-cc-cl   59.720     122.070
nd-ss-ss   65.090     97.440
ss-cc-nf   63.090     126.000
nf-ca-ca   67.850     120.610
ca-ca-ca   66.620     120.020
ca-ca-f    67.090     118.960
ca-ca-ha   48.180     119.880
ca-ca-cl   57.870     119.390

DIHE
cc-cc-nd-ss   1    4.750      180.000      2.000
cc-ss-ss-nd   1    0.000      0.000        3.000
cc-nf-ca-ca   1    0.000      180.000      3.000
cc-cc-ss-ss   1    1.100      180.000      2.000      same as X -c2-ss-X
cc-cc-nf-ca   1    4.150      180.000      2.000      same as X -c2-nf-X
cc-nd-ss-ss   1    4.800      180.000      2.000
nd-cc-cc-ss   1    4.000      180.000      2.000
nd-cc-cc-nf   1    4.000      180.000      2.000
ss-nd-cc-cl   1    4.750      180.000      2.000
ss-ss-cc-nf   1    1.100      180.000      2.000      same as X -c2-ss-X
ss-cc-cc-cl   1    4.000      180.000      2.000
ss-cc-nf-ca   1    4.150      180.000      2.000      same as X -c2-nf-X
cl-cc-cc-nf   1    4.000      180.000      2.000
nf-ca-ca-ca   1    3.625      180.000      2.000
nf-ca-ca-f    1    3.625      180.000      2.000
nf-ca-ca-ha   1    3.625      180.000      2.000
ca-ca-ca-ha   1    3.625      180.000      2.000
ca-ca-ca-ca   1    3.625      180.000      2.000
ca-ca-ca-cl   1    3.625      180.000      2.000
ha-ca-ca-f    1    3.625      180.000      2.000
ha-ca-ca-ha   1    3.625      180.000      2.000
f -ca-ca-ca   1    3.625      180.000      2.000
ha-ca-ca-cl   1    3.625      180.000      2.000

IMPROPER
cc-nf-cc-ss       1.1       180.0      2.0      Using default value
cc-cl-cc-nd       1.1       180.0      2.0      Using default value
ca-ca-ca-nf       1.1       180.0      2.0      Using default value
ca-ca-ca-f        1.1       180.0      2.0
ca-ca-ca-ha       1.1       180.0      2.0      General improper
torsional angle (2 general atom types)
ca-ca-ca-cl       1.1       180.0      2.0

NONBON
  cc        1.9080  0.0860
  nd        1.8240  0.1700
  ss        2.0000  0.2500
  cl        1.9480  0.2650
  nf        1.8240  0.1700
  ca        1.9080  0.0860
  ha        1.4590  0.0150
  f         1.7500  0.0610
```

I2.prepc

```
   0    0    2

This is a remark line
molecule.res
MOL   INT  0
CORRECT     OMIT DU   BEG
  0.0000
   1  DUMM  DU    M    0  -1  -2     0.000      .0       .0      .00000
   2  DUMM  DU    M    1   0  -1     1.449      .0       .0      .00000
   3  DUMM  DU    M    2   1   0     1.523   111.21      .0      .00000
   4  S     ss    M    3   2   1     1.540   111.208  -180.000  0.000000
   5  S1    ss    E    4   3   2     2.085    34.953   -58.452  0.000000
   6  N     nd    M    4   3   2     1.669    69.810    80.742  0.000000
   7  C1    cc    M    6   4   3     1.247   118.002   -22.574  0.000000
   8  Cl    cl    E    7   6   4     1.716   120.545  -179.947  0.000000
   9  C     cc    M    7   6   4     1.498   121.286     0.256  0.000000
  10  N1    nf    M    9   7   6     1.243   121.891   178.493  0.000000
  11  C2    ca    M   10   9   7     1.406   122.820   179.628  0.000000
  12  C3    ca    M   11  10   9     1.389   119.563   -45.036  0.000000
  13  HC    ha    E   12  11  10     1.103   120.060    -2.720  0.000000
  14  C4    ca    M   12  11  10     1.382   119.814   177.265  0.000000
  15  Cl1   cl    E   14  12  11     1.740   119.232   179.179  0.000000
  16  C5    ca    M   14  12  11     1.384   121.217    -1.267  0.000000
  17  H1    ha    E   16  14  12     1.103   120.473  -179.863  0.000000
  18  C6    ca    M   16  14  12     1.384   119.091     0.172  0.000000
  19  H2    ha    E   18  16  14     1.103   120.180  -179.558  0.000000
  20  C7    ca    M   18  16  14     1.376   119.602     0.451  0.000000
  21  F     f     M   20  18  16     1.327   119.229  -179.691  0.000000


LOOP
   C   S1
  C7   C2

IMPROPER
   C   Cl   C1    N
  C1   N1    C   S1
  C3   C7   C2   N1
  C2   C4   C3   HC
  C3   C5   C4  Cl1
  C4   C6   C5   H1
  C5   C7   C6   H2
  C2   C6   C7    F

DONE
STOP
```

I3.frcmod

```
remark goes here
MASS
cc 12.010        0.360
cd 12.010        0.360
nc 14.010        0.530
c  12.010        0.616
o  16.000        0.434
nh 14.010        0.530
c3 12.010        0.878
h1 1.008         0.135
os 16.000        0.465
ha 1.008         0.135
cl 35.450        1.910
ss 32.060        2.900

BOND
cc-cd  500.90   1.373
cc-ha  349.10   1.084
cc-cc  419.80   1.428
cd-cd  419.80   1.428
cd-ha  349.10   1.084
cd-nc  525.40   1.317
cd-nh  435.20   1.373
nc-c   416.90   1.387
c -o   637.70   1.218
c -cc  371.00   1.468
nh-c3  326.60   1.464
c3-c3  300.90   1.538
c3-h1  330.60   1.097
c3-os  308.60   1.432
cd-ss  265.80   1.756
cd-cl  317.50   1.735
nc-ss  359.80   1.626
ss-ss  153.80   2.073

ANGLE
cc-cd-cd   68.150     114.190
cc-cd-ha   48.540     121.760
cc-cc-c    63.620     122.690
cc-cc-cd   68.150     114.190
cd-cc-ha   48.540     121.760
cd-cd-nc   71.570     112.560
cd-cd-nh   68.170     119.720
cd-cd-ha   47.150     121.070
cd-nc-c    66.680     120.490
cd-nh-c3   63.700     119.720
nc-cd-nh   72.430     120.650
nc-c -o    73.910     123.180
nc-c -cc   68.580     113.750
c -cc-cd   65.090     121.350
o -c -cc   69.140     123.930
nh-c3-c3   66.210     110.460
nh-c3-h1   49.570     109.790
c3-nh-c3   63.250     114.510
c3-c3-h1   46.390     109.560
c3-c3-os   68.000     107.970
c3-os-c3   62.700     112.480
h1-c3-os   50.800     109.780
h1-c3-h1   39.240     108.460
ha-cc-cc   47.150     121.070
cc-cd-ss   64.850     111.550
cd-cd-cl   57.800     119.990
cd-ss-ss   63.530      93.800
cd-cd-ss   61.930     120.210
cd-nc-ss   68.550     108.070
cl-cd-nc   59.720     122.070
nc-ss-ss   65.090      97.440

DIHE
cc-cd-cd-nc   1    4.000       180.000           2.000
cc-cd-cd-nh   1    4.000       180.000           2.000
cc-cc-c -nc   1    2.875       180.000           2.000
cc-cc-c -o    1    2.875       180.000           2.000
cc-cc-cd-cd   1    4.000       180.000           2.000
cc-cc-cd-ss   1    4.000       180.000           2.000
cd-cc-cc-c    1    4.000       180.000           2.000
cd-cc-cc-cd   1    4.000       180.000           2.000
cd-cd-nc-c    1    4.750       180.000           2.000
cd-cd-nh-c3   1    1.050       180.000           2.000
cd-cd-cc-ha   1    4.000       180.000           2.000
cd-nc-c -o    1    4.000       180.000           2.000
cd-nc-c -cc   1    4.000       180.000           2.000
cd-nh-c3-c3   1    0.000         0.000           2.000
cd-nh-c3-h1   1    0.000         0.000           2.000
nc-cd-cd-ha   1    4.000       180.000           2.000
nc-cd-nh-c3   1    1.050       180.000           2.000
nc-c -cc-cd   1    2.875       180.000           2.000
c -nc-cd-nh   1    4.750       180.000           2.000
c -cc-cc-ha   1    4.000       180.000           2.000
c -cc-cd-cd   1    4.000       180.000           2.000
c -cc-cd-ss   1    4.000       180.000           2.000
o -c -cc-cd   1    2.875       180.000           2.000
nh-cd-cd-ha   1    4.000       180.000           2.000
nh-c3-c3-h1   1    0.156         0.000           3.000
nh-c3-c3-os   1    0.156         0.000           3.000
c3-nh-c3-c3   1    0.000         0.000           2.000
c3-nh-c3-h1   1    0.000         0.000           2.000
c3-c3-os-c3   1    0.383         0.000          -3.000
c3-c3-os-c3   1    0.100       180.000           2.000
c3-os-c3-h1   1    0.383         0.000           3.000
h1-c3-c3-h1   1    0.156         0.000           3.000
os-c3-c3-h1   1    0.000         0.000          -3.000
os-c3-c3-h1   1    0.250         0.000           1.000
ha-cd-cc-ha   1    4.000       180.000           2.000
ha-cd-cc-cc   1    4.000       180.000           2.000
ha-cc-cc-cd   1    4.000       180.000           2.000
cc-cd-cd-cl   1    4.000       180.000           2.000
cc-cd-ss-ss   1    1.100       180.000           2.000      same as X -c2-ss-X
cd-cd-nc-ss   1    4.750       180.000           2.000
cd-ss-ss-nc   1    0.000         0.000           3.000
cd-cd-ss-ss   1    1.100       180.000           2.000      same as X -c2-ss-X
cd-nc-ss-ss   1    4.800       180.000           2.000
cl-cd-cd-ss   1    4.000       180.000           2.000
cl-cd-nc-ss   1    4.750       180.000           2.000
nc-cd-cd-ss   1    4.000       180.000           2.000

IMPROPER
cc-cd-cc-ha         1.1         180.0         2.0         Using default value
cc-cd-cd-ha         1.1         180.0         2.0         Using default value
cd-nc-cd-nh         1.1         180.0         2.0         Using default value
cc-nc-c -o         10.5         180.0         2.0         General improper
torsional angle (2 general atom types)
c3-c3-nh-cd         1.1         180.0         2.0         Using default value
c -cc-cc-cd         1.1         180.0         2.0         Using default value
cc-cd-cd-ss         1.1         180.0         2.0         Using default value
cd-cl-cd-nc         1.1         180.0         2.0         Using default value

NONBON
  cc          1.9080  0.0860
  cd          1.9080  0.0860
  nc          1.8240  0.1700
  c           1.9080  0.0860
  o           1.6612  0.2100
  nh          1.8240  0.1700
  c3          1.9080  0.1094
  h1          1.3870  0.0157
  os          1.6837  0.1700
  ha          1.4590  0.0150
  cl          1.9480  0.2650
  ss          2.0000  0.2500
```

I3.prepc

```
   0    0    2

This is a remark line
molecule.res
MOL   XYZ  0
CHANGE     OMIT DU   BEG
  0.0000
   1  DUMM  DU    M       999.000     999.0      -999.0          .00000
   2  DUMM  DU    M       999.000    -999.0       999.0          .00000
   3  DUMM  DU    M      -999.000     999.0       999.0          .00000
   4  C1    cc    M         0.130000    1.277000   -0.264000   -0.150578
   5  C3    cd    B        -1.202000    1.283000   -0.251000   -0.422712
   6  C5    cd    B        -1.924000    0.031000   -0.024000    0.724084
   7  N1    nc    S        -1.321000   -1.107000    0.101000   -0.789439
   8  C4    c     S         0.036000   -1.192000    0.049000    0.828266
   9  O1    o     E         0.592000   -2.261000    0.122000   -0.576642
  10  N2    nh    S        -3.267000    0.054000    0.035000   -0.265217
  11  C6    c3    3        -4.019000   -1.195000    0.139000   -0.083045
  12  C8    c3    3        -5.345000   -1.050000   -0.589000    0.297255
  13  H4    h1    E        -5.928000   -1.954000   -0.466000    0.016767
  14  O2    os    S        -6.121000    0.026000   -0.119000   -0.517947
  15  C9    c3    3        -5.436000    0.988000    0.625000    0.297255
  16  C7    c3    B        -4.055000    1.283000    0.060000   -0.083045
  17  H3    h1    E        -3.583000    2.023000    0.694000    0.078671
  18  H7    h1    E        -4.153000    1.702000   -0.938000    0.078671
  19  H5    h1    E        -6.033000    1.889000    0.602000    0.016767
  20  H8    h1    E        -5.344000    0.676000    1.662000    0.016767
  21  H9    h1    E        -5.159000   -0.912000   -1.650000    0.016767
  22  H2    h1    E        -3.441000   -1.986000   -0.308000    0.078671
  23  H10   h1    E        -4.176000   -1.457000    1.181000    0.078671
  24  H1    ha    E        -1.734000    2.192000   -0.440000    0.178768
  25  H6    ha    E         0.637000    2.193000   -0.458000    0.208292
  26  C2    cc    M         0.873000    0.043000   -0.070000   -0.020293
  27  C10   cd    M         2.226000   -0.099000   -0.025000   -0.184152
  28  C11   cd    M         3.293000    0.945000   -0.025000    0.488368
  29  Cl1   cl    E         2.959000    2.648000    0.023000   -0.124188
  30  N3    nc    M         4.508000    0.645000   -0.017000   -0.406692
  31  S1    ss    M         4.909000   -0.959000    0.007000    0.118332
  32  S2    ss    M         2.963000   -1.699000    0.062000    0.101577


LOOP
   C2   C4
   C7   N2
   S2  C10

IMPROPER
   C2   C3   C1   H6
   C1   C5   C3   H1
   C3   N1   C5   N2
   C2   N1   C4   O1
   C6   C7   N2   C5
   C4   C1   C2  C10
   C2  C11  C10   S2
  C10  Cl1  C11   N3

DONE
STOP
```

Input structures

T-I-H2O

N	49.419	54.162	48.573
C	49.557	54.916	49.853
H	50.246	54.379	50.517
H	48.592	54.984	50.366
C	50.139	56.288	49.475
H	50.735	56.731	50.279
H	49.329	56.984	49.228
C	50.957	55.961	48.219
H	51.902	55.474	48.493
H	51.192	56.823	47.591
C	50.046	54.957	47.486
H	49.292	55.515	46.916
C	50.833	54.161	46.426
O	51.082	54.691	45.347
N	51.261	52.916	46.782
H	51.002	52.594	47.705
C	52.114	52.085	45.931
H	53.139	52.055	46.324
C	51.597	50.648	45.792
H	51.526	50.168	46.775
H	50.590	50.647	45.353
O	52.494	49.872	45.010
H	52.346	50.114	44.064
C	49.498	45.891	53.303
H	50.017	45.000	52.934
H	49.786	46.047	54.348
C	47.999	45.764	53.153
C	47.440	44.936	52.171
H	48.095	44.351	51.529
C	46.056	44.854	52.003
H	45.644	44.197	51.241
C	45.203	45.601	52.819
H	44.125	45.534	52.694
C	45.748	46.422	53.811
H	45.094	47.000	54.459
C	47.132	46.498	53.975
H	47.547	47.139	54.749
N	45.671	45.897	48.369
H	45.051	45.837	49.174
C	46.825	45.016	48.568
H	47.557	45.373	49.304
H	46.477	44.030	48.900
C	47.606	44.789	47.253
O	48.776	44.414	47.250
N	46.901	45.001	46.103
H	45.958	45.357	46.217
C	47.494	44.794	44.788
H	46.832	45.211	44.022
H	48.454	45.315	44.709
C	47.739	43.318	44.484
O	48.239	42.929	43.452
C	49.593	43.249	40.109
H	48.898	42.815	40.836
H	50.254	42.445	39.760
C	50.389	44.379	40.739
C	50.857	44.293	42.059
H	50.606	43.423	42.657
C	51.625	45.313	42.634
H	51.982	45.218	43.657
C	51.932	46.451	41.885
O	52.672	47.512	42.376
H	53.070	47.322	43.244
C	51.467	46.565	40.575
H	51.702	47.459	40.006
C	50.706	45.539	40.018
H	50.352	45.644	38.995
C	56.107	48.946	45.609
H	56.904	48.220	45.419
H	56.065	49.126	46.696
O	54.901	48.395	45.099
H	54.169	49.027	45.243
C	51.978	50.933	41.289
H	53.029	50.884	40.969
H	51.741	51.965	41.559
O	51.730	50.132	42.440
H	51.959	49.197	42.253
C	43.067	51.462	49.793
H	42.448	51.824	48.966
C	44.537	51.371	49.378
H	45.151	50.989	50.198
H	44.663	50.687	48.533
S	45.266	52.977	48.823
H	45.420	53.492	50.064
C	48.118	48.480	46.703
C	49.156	47.646	46.262
C	49.564	47.693	44.931
H	50.375	47.071	44.568
H	49.618	46.947	46.951
N	47.692	48.427	48.054
C	47.899	49.435	48.811
C	47.475	49.490	50.233
N	47.773	50.485	50.988
S	48.629	51.739	50.299
S	48.745	50.948	48.330
C	47.480	49.330	45.789
H	46.666	49.966	46.128
C	47.863	49.357	44.448
H	47.346	50.008	43.756
C	48.911	48.536	44.031
O	49.378	48.450	42.724
C	48.835	49.204	41.741
F	49.509	48.948	40.617
F	48.898	50.526	41.986
F	47.534	48.905	41.531

H	49.874	46.750	52.730
H	47.418	42.615	45.282
H	46.017	46.857	48.344
H	48.436	54.011	48.358
C	43.151	57.489	51.986
H	42.328	57.091	51.386
C	43.910	58.599	51.231
H	43.161	59.301	50.839
H	44.512	59.184	51.938
C	44.807	58.121	50.097
C	44.473	57.016	49.298
H	43.551	56.468	49.477
C	45.324	56.587	48.277
H	45.045	55.724	47.680
C	46.526	57.256	48.034
H	47.187	56.924	47.238
C	46.869	58.358	48.820
H	47.799	58.891	48.637
C	46.018	58.781	49.844
H	46.296	59.640	50.452
C	43.966	56.270	52.415
O	43.411	55.172	52.565
H	49.018	43.601	39.246
H	51.333	50.634	40.454
H	56.386	49.893	45.117
H	52.148	52.570	44.952
H	42.941	52.151	50.636
H	42.699	50.473	50.097
N	45.291	56.441	52.666
H	45.774	57.248	52.302
H	45.843	55.598	52.792
H	42.694	57.908	52.891
Cl	46.562	48.170	50.884
O	45.553	53.497	52.241
H	44.653	53.827	52.459
H	45.655	52.625	52.641

TS1-A-H2O

C	49.103	53.666	49.190
H	49.882	52.897	49.247
H	48.178	53.243	49.591
C	49.561	54.958	49.898
H	50.166	54.755	50.787
H	48.684	55.532	50.222
C	50.346	55.729	48.804
H	51.424	55.749	48.984
H	50.014	56.770	48.731
C	50.046	54.957	47.486
H	49.770	55.626	46.664
C	51.307	54.185	46.991
O	52.398	54.740	46.946
N	51.097	52.890	46.610
H	50.127	52.595	46.597
C	52.114	52.085	45.931
H	53.090	52.309	46.367
C	51.788	50.584	46.023
H	52.128	50.172	46.977
H	50.702	50.432	45.954
O	52.447	49.826	45.003
H	52.199	50.207	44.130
C	49.498	45.891	53.303
H	48.696	45.268	53.712
H	49.989	46.391	54.148
C	48.961	46.909	52.315
C	47.669	47.439	52.452
H	47.031	47.084	53.259
C	47.192	48.421	51.578
H	46.189	48.820	51.713
C	48.001	48.895	50.542
H	47.636	49.659	49.859
C	49.286	48.365	50.383
H	49.926	48.723	49.580
C	49.755	47.381	51.259
H	50.757	46.980	51.126
N	46.802	46.483	48.547
H	46.273	46.840	49.339
C	46.825	45.016	48.568
H	47.488	44.569	49.321
H	45.812	44.650	48.772
C	47.248	44.413	47.212
O	47.676	43.267	47.133
N	47.095	45.224	46.118
H	46.733	46.159	46.270
C	47.494	44.794	44.788
H	47.837	45.647	44.199
H	48.326	44.086	44.896
C	46.399	44.077	44.013
O	46.455	43.894	42.815
C	49.593	43.249	40.109
H	48.547	43.123	40.409
H	50.114	42.309	40.335
C	50.226	44.414	40.842
C	51.141	45.270	40.215
H	51.389	45.113	39.169
C	51.730	46.335	40.896
H	52.429	47.001	40.400
C	51.407	46.564	42.234
O	51.994	47.664	42.838
H	51.843	47.684	43.800
C	50.501	45.726	42.886
H	50.239	45.910	43.926
C	49.919	44.668	42.186
H	49.183	44.042	42.680
C	56.107	48.946	45.609
H	56.996	49.102	46.229
H	56.398	49.107	44.557
O	55.118	49.855	46.054
H	54.307	49.737	45.524
C	51.978	50.933	41.289
H	53.029	50.644	41.152
H	51.925	52.016	41.426
O	51.409	50.331	42.452
H	51.521	49.357	42.388
C	43.064	51.443	49.729
H	42.823	52.383	50.238
C	44.577	51.246	49.613
H	45.061	51.227	50.594
H	44.810	50.314	49.091
S	45.399	52.620	48.682
H	44.432	52.688	47.706
C	47.414	48.106	42.862
C	48.493	48.887	42.390
C	49.012	48.655	41.123
H	49.822	49.262	40.740
H	48.930	49.683	42.979
N	46.697	48.290	44.038
C	46.803	49.199	44.937
C	45.701	49.350	45.917
N	45.585	50.342	46.728
S	46.869	51.463	46.893
S	48.017	50.479	45.191
C	46.935	47.064	42.038
H	46.119	46.452	42.402
C	47.474	46.809	40.784
H	47.095	45.987	40.192
C	48.502	47.633	40.325
O	49.122	47.512	39.085
C	48.536	46.816	38.080
F	49.278	46.993	36.988
F	47.282	47.234	37.831
F	48.470	45.490	38.333
H	50.238	45.232	52.837
H	45.540	43.723	44.622
H	47.744	46.846	48.679
H	48.044	54.524	47.669
C	43.151	57.489	51.986
H	42.323	56.981	51.478
C	43.932	58.362	50.969
H	43.242	59.127	50.589
H	44.728	58.894	51.503
C	44.522	57.592	49.803
C	43.691	56.916	48.894
H	42.611	56.963	49.018
C	44.229	56.197	47.825
H	43.568	55.684	47.131
C	45.614	56.150	47.637
H	46.029	55.607	46.791
C	46.451	56.819	48.531
H	47.529	56.803	48.391
C	45.908	57.527	49.608
H	46.569	58.042	50.301
C	44.051	56.467	52.676
O	44.539	56.671	53.783
H	49.622	43.396	39.026
H	51.418	50.671	40.381
H	55.802	47.890	45.716
H	52.164	52.366	44.868
H	42.636	50.620	50.316
H	42.595	51.445	48.743
N	44.276	55.301	51.985
H	44.110	55.256	50.989
H	45.001	54.699	52.353
H	42.736	58.128	52.769
Cl	44.385	48.213	45.870
O	43.479	51.758	46.549
H	44.225	51.073	46.600
H	43.262	51.886	45.618

INT1A-H2O

N	49.857	54.149	48.710
C	50.849	54.672	49.668
H	51.820	54.218	49.432
H	50.585	54.383	50.690
C	50.902	56.194	49.433
H	51.856	56.637	49.737
H	50.115	56.686	50.018
C	50.633	56.337	47.914

H	51.543	56.546	47.345
H	49.931	57.149	47.701
C	50.046	54.957	47.486
H	49.087	55.069	46.968
C	50.983	54.241	46.469
O	51.451	54.859	45.517
N	51.204	52.919	46.714
H	50.796	52.568	47.575
C	52.114	52.085	45.931
H	53.063	51.933	46.462
C	51.497	50.712	45.628
H	51.187	50.232	46.565
H	50.595	50.833	45.011
O	52.426	49.828	45.007
H	52.472	50.035	44.047
C	49.498	45.891	53.303
H	50.089	44.994	53.093
H	49.481	46.026	54.393
C	48.095	45.774	52.751
C	47.502	44.522	52.537
H	48.075	43.620	52.741
C	46.188	44.416	52.074
H	45.748	43.432	51.925
C	45.441	45.567	51.811
H	44.421	45.498	51.444
C	46.025	46.820	52.012
H	45.457	47.724	51.806
C	47.337	46.921	52.474
H	47.782	47.903	52.621
N	45.439	44.564	48.402
H	45.107	44.175	49.282
C	46.825	45.016	48.568
H	46.968	45.891	49.210
H	47.399	44.196	49.017
C	47.497	45.334	47.217
O	48.461	46.092	47.141
N	46.962	44.688	46.137
H	46.134	44.132	46.328
C	47.494	44.794	44.788
H	46.718	45.042	44.058
H	48.223	45.614	44.791
C	48.220	43.529	44.364
O	48.342	43.172	43.212
C	49.593	43.249	40.109
H	48.734	43.328	40.780
H	50.119	42.322	40.375
C	50.505	44.448	40.242
C	51.210	44.952	39.143
H	51.085	44.488	38.167
C	52.067	46.046	39.266
H	52.591	46.434	38.395
C	52.228	46.655	40.510
O	53.059	47.748	40.688
H	53.436	48.008	39.833
C	51.543	46.172	41.624
H	51.675	46.658	42.585
C	50.686	45.082	41.480
H	50.140	44.716	42.344
C	56.107	48.946	45.609
H	57.092	49.231	45.994
H	56.201	48.803	44.518
O	55.205	49.980	45.955
H	54.310	49.764	45.627
C	51.978	50.933	41.289
H	52.878	51.462	40.942
H	51.309	51.661	41.756
O	52.296	49.950	42.269
H	52.627	49.152	41.811
C	43.064	51.443	49.729
H	43.232	51.994	50.661
C	44.325	50.727	49.264
H	44.689	50.035	50.025
H	44.126	50.166	48.350
S	45.677	51.944	48.916
H	42.770	47.517	49.272
C	47.369	48.261	44.376
C	48.765	48.430	44.459
C	49.518	48.591	43.302
H	50.587	48.758	43.353
H	49.266	48.389	45.419
N	46.533	48.056	45.475
C	46.604	48.591	46.624
C	45.647	48.101	47.660
N	45.913	47.962	48.867
S	47.369	50.729	48.937
S	47.754	49.956	47.025
C	46.764	48.192	43.110
H	45.694	48.016	43.055
C	47.508	48.366	41.947
H	47.019	48.338	40.983
C	48.884	48.572	42.059
O	49.731	48.789	40.974
C	49.347	48.493	39.712
F	50.409	48.701	38.922
F	48.346	49.289	39.273
F	48.938	47.224	39.573
H	50.025	46.751	52.876
H	48.658	42.946	45.205
H	44.837	45.361	48.201
H	48.918	54.288	49.076
C	43.151	57.489	51.986
H	42.459	56.757	51.552
C	43.908	58.225	50.849
H	43.165	58.762	50.246
H	44.562	58.983	51.295
C	44.718	57.302	49.960
C	44.077	56.356	49.142
H	42.989	56.322	49.115
C	44.815	55.465	48.360
H	44.304	54.740	47.732
C	46.213	55.511	48.374
H	46.782	54.817	47.761
C	46.861	56.456	49.170
H	47.945	56.512	49.178
C	46.118	57.339	49.961
H	46.632	58.065	50.586
C	44.101	56.802	52.962
O	44.455	57.331	54.011
H	49.229	43.137	39.082
H	51.465	50.488	40.431
H	55.840	47.972	46.054
H	52.334	52.627	45.007
H	42.270	50.707	49.907
H	42.695	52.149	48.976
N	44.529	55.549	52.596
H	44.466	55.259	51.630
H	45.288	55.161	53.141
H	42.571	58.213	52.563
Cl	43.983	47.710	46.949
O	43.221	47.366	50.115
H	45.155	47.623	49.464
H	42.628	47.677	50.811

TS2A-H2O

N	49.399	54.018	48.43
C	50.156	54.171	49.687
H	51.084	53.590	49.598
H	49.583	53.768	50.526
C	50.474	55.674	49.799
H	51.346	55.880	50.429
H	49.616	56.194	50.244
C	50.681	56.110	48.328
H	51.737	56.218	48.069
H	50.205	57.072	48.114
C	50.046	54.957	47.486
H	49.303	55.332	46.774
C	51.135	54.254	46.627
O	51.923	54.917	45.959
N	51.139	52.894	46.666
H	50.453	52.464	47.278
C	52.114	52.085	45.931
H	53.083	52.076	46.447
C	51.610	50.658	45.762
H	51.463	50.187	46.742
H	50.639	50.666	45.25
O	52.544	49.866	45.047
H	52.421	50.079	44.093
C	49.498	45.891	53.303
H	49.692	46.895	52.905
H	50.023	45.178	52.657
C	48.015	45.607	53.333
C	47.300	45.469	52.133
H	47.829	45.568	51.19
C	45.929	45.215	52.13
H	45.398	45.133	51.188
C	45.238	45.092	53.342
H	44.171	44.892	53.346
C	45.935	45.232	54.548
H	45.411	45.134	55.495
C	47.313	45.489	54.539
H	47.846	45.593	55.481
N	45.775	46.019	48.401
H	44.854	45.589	48.349
C	46.825	45.016	48.568
H	47.699	45.513	49.001
H	46.579	44.160	49.212
C	47.307	44.431	47.218
O	47.938	43.381	47.161
N	47.017	45.178	46.11
H	46.511	46.043	46.267
C	47.494	44.794	44.788
H	47.422	45.651	44.111
H	48.549	44.499	44.844
C	46.725	43.618	44.194
O	46.982	43.141	43.111
C	49.593	43.249	40.109
H	48.736	42.883	40.681
H	50.246	42.395	39.889
C	50.332	44.329	40.873

C	51.511	44.913	40.386
H	51.925	44.578	39.437
C	52.162	45.933	41.086
H	53.060	46.394	40.682
C	51.644	46.376	42.306
O	52.192	47.447	43.005
H	53.162	47.385	43.061
C	50.490	45.786	42.821
H	50.109	46.118	43.778
C	49.841	44.783	42.102
H	48.936	44.332	42.496
C	56.107	48.946	45.609
H	56.973	48.361	45.282
H	56.007	48.822	46.7
O	54.976	48.464	44.899
H	54.194	48.990	45.159
C	51.978	50.933	41.289
H	52.959	50.650	40.881
H	51.992	52.000	41.523
O	51.678	50.245	42.503
H	51.655	49.278	42.364
C	43.064	51.443	49.729
H	42.661	52.233	49.085
C	43.875	52.027	50.883
H	43.279	52.714	51.493
H	44.290	51.239	51.515
S	45.286	53.091	50.303
H	46.717	48.570	53.029
C	48.340	48.277	47.649
C	49.589	47.828	47.197
C	49.925	47.937	45.851
H	50.914	47.685	45.483
H	50.304	47.430	47.911
N	48.046	48.194	49.028
C	47.674	49.222	49.689
C	47.410	48.998	51.1
N	47.589	48.870	52.252
S	47.058	52.121	50.745
S	47.689	50.927	49.086
C	47.393	48.747	46.726
H	46.406	49.037	47.072
C	47.711	48.831	45.372
H	46.983	49.214	44.668
C	48.990	48.456	44.956
O	49.466	48.611	43.658
C	48.627	48.681	42.601
F	49.368	48.628	41.494
F	47.913	49.826	42.579
F	47.739	47.662	42.582
H	49.942	45.835	54.302
H	45.900	43.226	44.826
H	45.751	46.687	49.169
H	48.437	54.312	48.592
C	43.151	57.489	51.986
H	42.226	57.465	51.407
C	44.263	58.257	51.233
H	43.829	59.195	50.861
H	45.049	58.562	51.936
C	44.901	57.490	50.085
C	44.133	56.712	49.205
H	43.058	56.638	49.343
C	44.735	56.009	48.16
H	44.122	55.406	47.495
C	46.117	56.074	47.97
H	46.584	55.532	47.151
C	46.892	56.848	48.835
H	47.966	56.920	48.691
C	46.287	57.544	49.885
H	46.901	58.145	50.554
C	43.487	56.027	52.293
O	42.679	55.121	52.104
H	49.221	43.625	39.147
H	51.206	50.747	40.53
H	56.317	50.009	45.404
H	52.275	52.550	44.953
H	42.223	50.870	50.138
H	43.671	50.760	49.129
N	44.725	55.784	52.828
H	45.462	56.465	52.719
H	45.020	54.817	52.824
H	42.926	58.002	52.929
Cl	44.862	48.559	50.71
O	45.598	48.294	53.494
H	45.575	47.363	53.791
H	45.086	48.337	52.613

TIA-H2O

N	49.369	54.016	48.408
C	50.117	54.131	49.675
H	51.030	53.529	49.587
H	49.525	53.730	50.503
C	50.470	55.625	49.816
H	51.348	55.796	50.448
H	49.627	56.156	50.275
C	50.685	56.088	48.354
H	51.742	56.195	48.100
H	50.215	57.056	48.158
C	50.046	54.957	47.486
H	49.321	55.352	46.767
C	51.138	54.247	46.637
O	51.946	54.906	45.991
N	51.125	52.886	46.656
H	50.429	52.451	47.254
C	52.114	52.085	45.931
H	53.077	52.082	46.458
C	51.631	50.648	45.734
H	51.518	50.138	46.699
H	50.652	50.650	45.237
O	52.572	49.901	44.972
H	52.464	50.202	44.039
C	49.498	45.891	53.303
H	49.679	46.897	53.704
H	50.002	45.830	52.332
C	48.017	45.625	53.172
C	47.299	46.104	52.067
H	47.823	46.666	51.297
C	45.926	45.884	51.950
H	45.391	46.281	51.094
C	45.238	45.181	52.942
H	44.168	45.017	52.858
C	45.939	44.704	54.053
H	45.415	44.162	54.836
C	47.315	44.924	54.163
H	47.852	44.548	55.030
N	45.454	45.474	48.325
H	44.839	45.183	49.080
C	46.825	45.016	48.568
H	47.402	45.626	49.272
H	46.799	43.998	48.973
C	47.650	44.968	47.264
O	48.875	45.059	47.278
N	46.914	44.805	46.123
H	45.908	44.848	46.253
C	47.494	44.794	44.788
H	47.048	45.560	44.146
H	48.562	45.025	44.898
C	47.376	43.439	44.111
O	47.186	43.290	42.924
C	49.593	43.249	40.109
H	48.766	42.732	40.610
H	50.393	42.524	39.929
C	50.077	44.415	40.946
C	51.430	44.614	41.242
H	52.168	43.905	40.874
C	51.866	45.706	42.003
H	52.924	45.841	42.217
C	50.933	46.625	42.482
O	51.283	47.746	43.212
H	52.184	47.678	43.576
C	49.573	46.442	42.210
H	48.852	47.150	42.596
C	49.159	45.349	41.453
H	48.098	45.201	41.276
C	56.107	48.946	45.609
H	56.939	48.276	45.368
H	56.034	49.010	46.708
O	54.942	48.409	45.011
H	54.192	49.008	45.196
C	51.978	50.933	41.289
H	52.724	50.251	40.856
H	52.432	51.920	41.405
O	51.553	50.515	42.582
H	51.165	49.620	42.536
C	43.064	51.443	49.729
H	42.636	52.133	48.995
C	43.702	52.201	50.893
H	42.985	52.872	51.376
H	44.110	51.524	51.648
S	45.048	53.363	50.377
H	45.673	48.618	53.484
C	47.072	48.869	46.918
C	48.251	48.215	46.539
C	48.806	48.468	45.287
H	49.734	48.005	44.972
H	48.729	47.509	47.211
N	46.525	48.623	48.208
C	46.493	49.575	49.063
C	45.902	49.300	50.354
N	45.426	49.087	51.391
S	46.809	52.283	50.568
S	47.208	51.218	48.779
C	46.420	49.727	46.022
H	45.494	50.211	46.316
C	46.965	49.974	44.763
H	46.471	50.652	44.078
C	48.163	49.351	44.420
O	48.834	49.595	43.219
C	48.146	49.825	42.077
F	49.012	49.732	41.064
F	47.586	51.052	42.042

F	47.159	48.929	41.887
H	49.976	45.176	53.980
H	47.491	42.572	44.799
H	45.444	46.493	48.295
H	48.412	54.329	48.566
C	43.151	57.489	51.986
H	42.257	57.499	51.359
C	44.329	58.205	51.288
H	43.968	59.188	50.953
H	45.121	58.423	52.016
C	44.932	57.453	50.113
C	44.127	56.790	49.173
H	43.048	56.779	49.294
C	44.697	56.126	48.085
H	44.055	55.615	47.372
C	46.083	56.114	47.912
H	46.525	55.605	47.059
C	46.894	56.770	48.840
H	47.971	56.786	48.710

C	46.321	57.426	49.933
H	46.964	57.937	50.647
C	43.415	56.023	52.320
O	42.628	55.132	52.004
H	49.224	43.587	39.132
H	51.127	51.013	40.601
H	56.361	49.950	45.229
H	52.281	52.555	44.956
H	42.260	50.800	50.107
H	43.789	50.804	49.213
N	44.559	55.757	53.018
H	45.296	56.443	53.080
H	44.836	54.787	53.083
H	42.902	58.023	52.912
O	45.917	48.391	54.393
H	46.107	47.444	54.362
